# Photon trapping in a time cavity flying at the speed of light

**DOI:** 10.1038/s41467-026-77059-1

**Published:** 2026-08-29

**Authors:** Ihar Babushkin, Oliver Melchert, Uwe Morgner, Ayhan Demircan

**Affiliations:** 1 Cluster of Excellence PhoenixD, Welfengarten 1, Hannover, Germany; 2 Institute of Quantum Optics, Leibniz Universität Hannover, Welfengarten 1, Hannover, Germany; 3 Max Born Institute for Nonlinear Optics and Short Pulse Spectroscopy, Rudower Chaussee 17, Berlin, Germany; 4 Hannover Centre for Optical Technologies, Nienburger Str.17, Hannover, Germany

**Keywords:** Ultrafast photonics, Solitons, Single photons and quantum effects

## Abstract

A strong optical pulse in a nonlinear medium can serve as a trap for photons. Photon confinement in such a trap moving at the speed of light is best described as time-trapping. Here we reveal unusual properties that fundamentally distinguish it from conventional trapping. Time-trapped states can exhibit zero leakage through the trap walls and possess extremely broad spectra while remaining genuine single modes with well-defined phase. Linear beatings between modes enable waveform reshaping in both the temporal and spectral domains. Time-trapped states support efficient coupling to external radiation, allowing fundamentally more efficient loading of energy than stationary cavities and surpassing the conventional Lorentz time-bandwidth limit linking lifetime and bandwidth in conventional resonant systems. Time-traps support exotic phenomena including halo states and anomalously large transmission delays. These properties apply equally to weak classical wavepackets and single-photon states, opening new opportunities for ultrafast classical and quantum photonics.

## Abstract (web summary)

A strong optical pulse can form a trap, moving at the speed of light and best described as a time trap. Here, the authors investigate time-trapping unveiling unusual properties, enabling efficient energy storage and manipulation of ultrafast classical and quantum light.

## Introduction

**Fig. 1:**
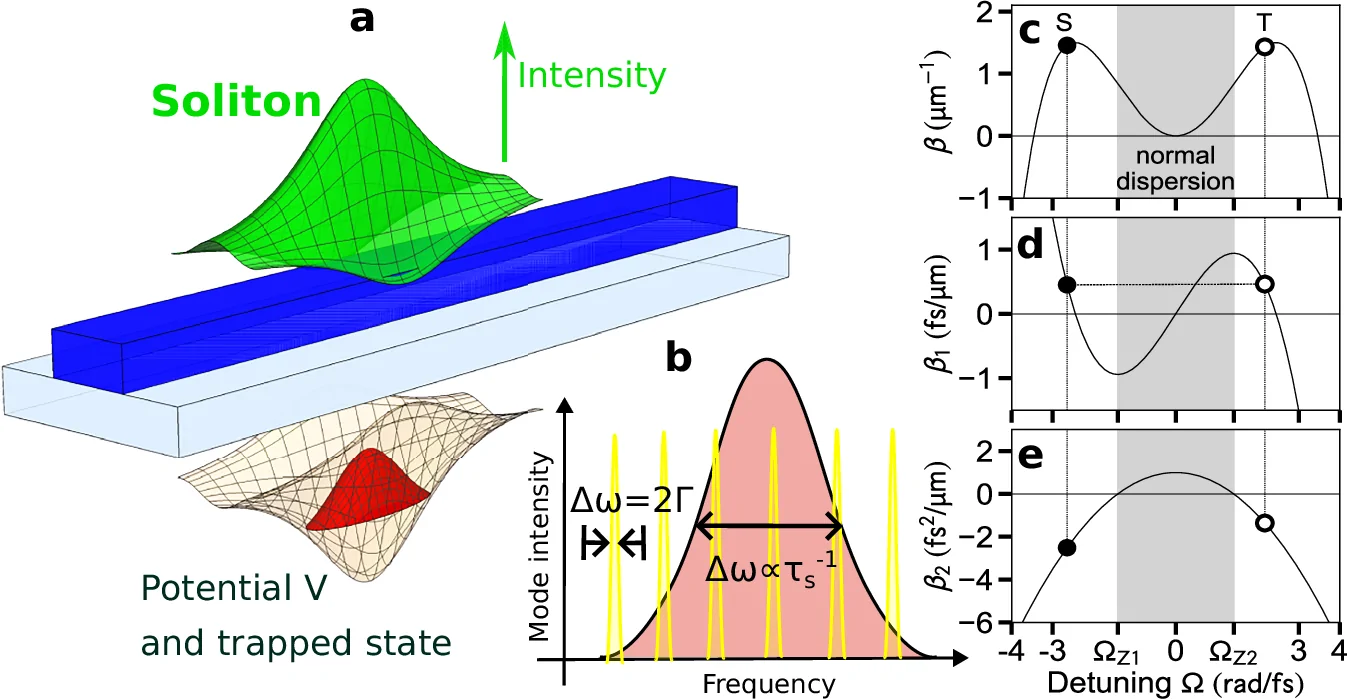
Time-trapping. **a** A waveguide (blue) with a soliton (green) creating an index step moving at the speed of light, building up an effective potential *V*(*t*) (light orange), best described as a “time-potential'', which serves as a trap for weak light waves or single photons (red). **b** Spectrally broad time-trapped mode (orange) vs. narrow normal cavity modes (yellow). **c**–**e** Exemplary dispersion curve allowing time-trapping in a single-mode waveguide: the trapped radiation (T) is group-matched with a soliton (S) and located in a separate anomalous range (two regions of anomalous dispersion are bounded by *Ω*_*Z*1_ and *Ω*_*z*2_). Here and thereafter, we denote by *Ω* the detuning from an arbitrarily selected frequency *ω*_0_: *Ω* = *ω* − *ω*_0_. All the corresponding frequencies *ω*_*s*_, *ω*_*t*_. …, defined in the text, are then converted to the corresponding detunings *Ω*_*S*_, *Ω*_*T*_, ….

In contrast to most other common particles surrounding us, photons have zero rest mass – this is why light is so difficult to trap. This, however, allows for an unusual type of confinement: in a trap moving at the speed of light. The most straightforward way to create such a trap is to use an optical soliton^1–3^, see Fig. 1a. Solitons are capable of propagating over extended, even astronomical distances^4^. They create a moving refractive-index inhomogeneity, a potential well, which can trap light at different frequency or mode^1–3^. The radiation to be trapped should typically be located in the anomalous dispersion range and group-velocity-matched to the soliton. This can be achieved using different (for instance, orthogonally polarized) modes^1,2^ or, alternatively, dispersion profiles with several anomalous ranges^5–8^, see Fig. 1c–e. The latter allows one to build multi-frequency “meta-atoms,” with the constituents located at incommensurate frequencies. The trapped states form a discrete spectrum, with different temporal waveforms^2,3^ as the eigenmodes. Trapping can also be realized in non-solitonic pulses, provided such pulses are located near zero dispersion point ^9^, or if they are “periodically renewed”^10^. Trapping increases photon localization, thereby boosting the nonlinear interaction^5,10^ and making such systems an attractive candidate for all-optical quantum gates^10^.

Such trapping is best described as “time-trapping” because the trapping potential – in the moving reference frame – is a time-dependent, rather than a space-dependent, function. Thus, it belongs to a new, actively growing field of “time-optics”, which also includes temporal analogs of reflection and refraction^11–16^, time interfaces^17^, time lenses^18–20^, time crystals^21–24^ and others. Time-optics can be induced using homogeneous modification of material properties^17^, but also, as in our situation, using optical pulses^1–3,6,7,12,14^. In time-optics, there are effects closely related to time-trapping: pushbroom effect^25,26^ (dispersive wave “pushed” by a soliton), trapping near the accelerating soliton^27^, refractive-index-front-induced light stopping and reversal^28,29^.

Does time trapping share the same fundamental properties and limitations as trapping in traditional cavities? Traditional cavities can demonstrate extremely large quality factors *Q* (as high as *Q* ≈ 10^9^^30^), yet at the price of extremely weak coupling to external radiation (large lifetime *Δt*) and extremely narrow linewidth *Δω* (for instance, kHz-scale) for their modes; these quantities are connected to each other via the time–bandwidth product *ΔωΔt*  which must be of the order of unity ^31–33^. This limitation is relevant far beyond optics; it also fundamentally limits the speed with which energy in the mode can be manipulated, in particular, stored from an external pulse. For slow-light configurations, such as those inspired by electromagnetically induced transparency^34,35^, these limitations are especially important. It was suggested^32^ that the time–bandwidth product can be broken in nonreciprocal slow-light cavities, but whether this is true or not is under debate^33^.

Here, we show that time-trapping provides a fundamentally different confinement regime in which these traditional limitations become irrelevant, allowing, in particular, the loading of external light into the trap fundamentally faster than is permitted in passive linear optics. Because of zero leakage through the walls of the time-trap, time-trapping promises *Q*-factors that surpass those of traditional cavities by orders of magnitude. Furthermore, time-traps confine extremely broadband (up to petahertz-scale) radiation as genuine modes, which, as we show, enable new ways to manipulate such modes that are impossible in traditional linear optics, as well as the realization of several exotic states and phenomena such as halo states and anomalously long transmission delays. We explicitly formulate our approach in a form that is equally well applicable to weak classical light and single-photon Fock-state waveshapes, discuss the applicability of time traps as photonic memories and demonstrate the influence of trapping dynamics on nonclassicality and photon statistics.

## Results

**Fig. 2:**
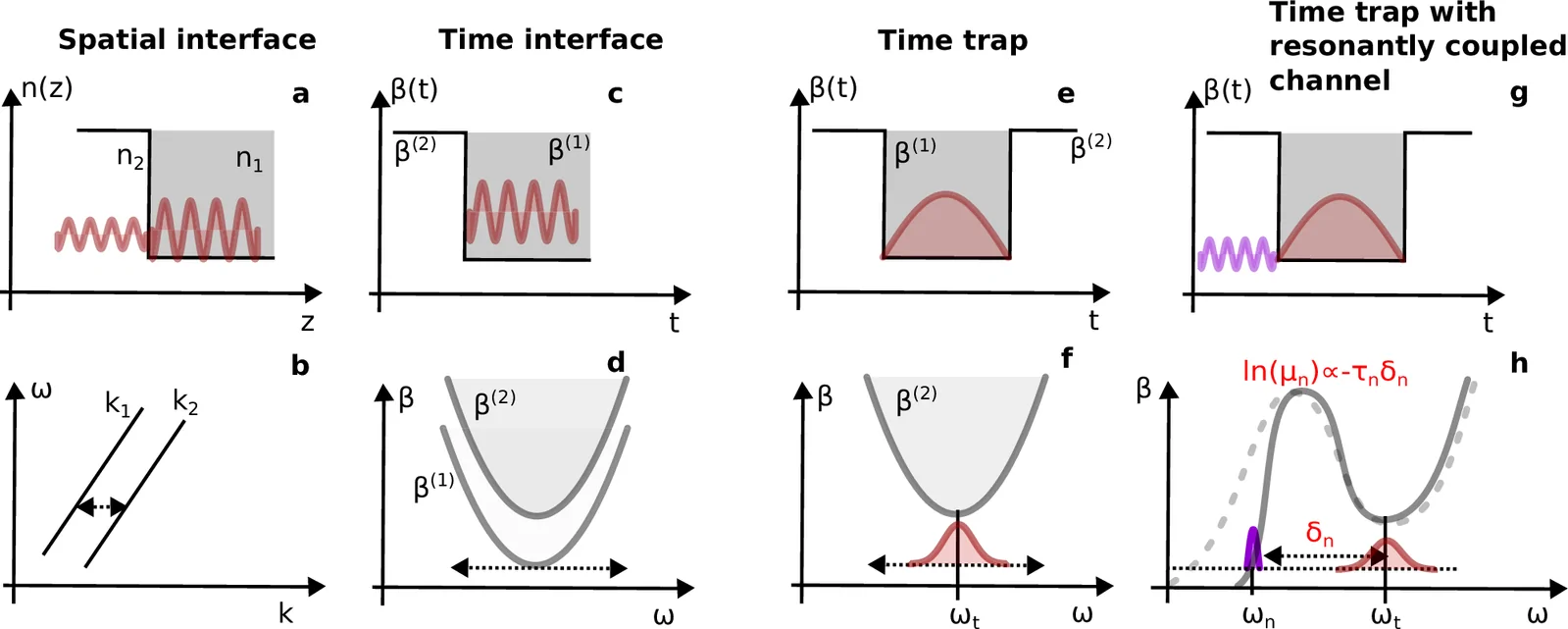
Mechanism of ideal light trapping in a time-trap. **a**–**d** Reflection at a standing (**a**, **b**) and a moving (**c**, **d**) interface ("time-interface'') in real space (**a**, **c**) and in wavevector or frequency space (**b**, **d**). *n*_*i*_, *k*_*i*_, and *β*^(*i*)^ denote the refractive indices and wavevectors on both sides of the interface, in the resting (*k*_*i*_) and moving (*β*^(*i*)^) reference frames, respectively. Dashed arrows indicate the presence or absence of a phase-matched wave on the opposite side of the interface. **e**–**h** Two such time-interfaces form a time-trap, giving rise to a discrete spectrum of trapped states that inherit the “no transmitted wave” property of the individual interfaces. **g**, **h** Proper tuning of higher-order dispersion allows the resonance—and therefore the emitted wave—to reappear, with coupling strength *μ*_*n*_ exponentially dependent on both the frequency mismatch *δ*_*n*_ and the trapped state duration *τ*_*n*_.

Our list of distinctive features of time-trapping begins with the observation that it can exhibit effectively zero radiation through the walls. Mathematically, one can foresee this already in the absence of imaginary parts in the eigenvalues of the trapped states^2,3^. To reveal the physical origin of this phenomenon, we recall that the presence or absence of the leakage at an interface (cavity wall) is determined by the presence or absence of phase-matched wavevectors (or frequencies) on the other side of the interface (see Fig. 2)–this is an alternative (but equivalent) reformulation of the continuity of the tangential components of the field at the boundary. In the case of traditional, standing cavities, we must search for matching wavevectors *k* for fixed *ω*. As soon as we are interested in ultrashort pulses and broad spectral ranges, we have *ω* ~ *k* + … in the leading order (the wavevector is mostly a linear function of *ω*), making the presence of the matching wavelengths and thus losses unavoidable (cf. Fig. 2a, b). For a moving interface, the situation is fundamentally different^12^: we must consider the field in the co-moving reference frame, with the modified wavevector *β* = *k*(*ω*) − *ω*/*v*_*g*_, where *v*_*g*_ is the group velocity of the interface, and search for matched *ω* for fixed *β* (cf. Fig. 2c, d)–meaning, in particular, that the leakage at a moving interface is simultaneously a frequency-conversion process. The linear term in *β*(*ω*) is fully canceled by the frame-of-reference transformation, so that the leading term can only be quadratic. If this is the only remaining term, phase matching at the interface is fully prohibited, meaning that no outgoing wave is possible anymore. As explained later, allowing higher-order dispersion can reopen a radiation channel in a tunable, controllable way (see Fig. 2g,h).

**Fig. 3:**
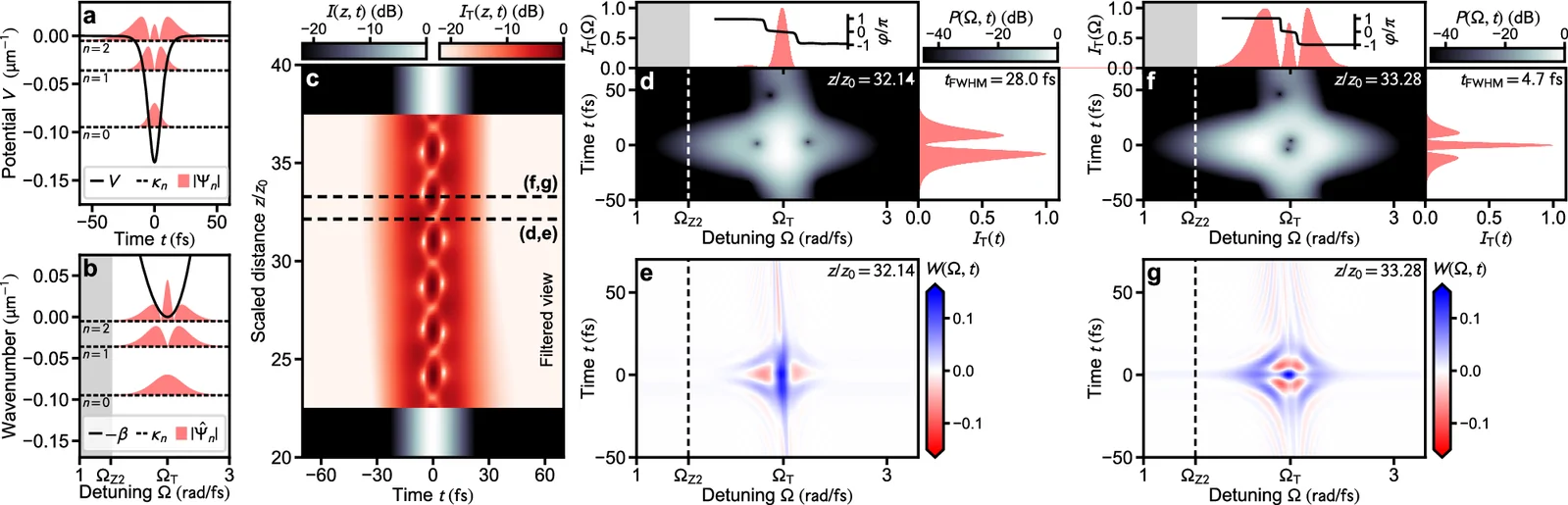
Beatings between time-modes leading to pulse shaping in both the time and frequency domains simultaneously. **a**, **b** A time-trap supporting multiple trapped modes (**a**) and their corresponding spectra (**b**). In **a**, the black line denotes the time-potential, whereas in **b** it denotes the dispersion −*β*(*ω*) (in the reference frame of the trap). **c** Temporal waveforms resulting from the beating of exemplary modes with *n* = 0 and *n* = 2; propagation distance *z* is normalized to *z*_0_ ≈ 30.6 μm. I_T_(z, t) is the intensity of the trapped state; The soliton intensity *I(z, t)* is shown using black-and-white color encoding, while the intensity of the trapped state is shown using red-and-black encoding. **d**–**g** Temporal and spectral intensity profiles (including phase information), along with the spectrogram *P*(*Ω*, *t*) (**d**, **f**) of weak classical states and Wigner functions *W*(*Ω*, *t*) (**e**, **g**) (see definitions of *P* and *W* in “Methods”) of single-photon states for two selected positions [marked in by dashed lines in (**c**)]. That is, starting from (**d**, **e**) and propagating to (**f**, **g**), these linear beatings produce Fourier-limited pulse shortening, with the FWHM duration reduced by approximately a factor of six.

Whereas the trapped modes of a good stationary cavity are well known to be located at different frequencies (see Fig. 1b) and have narrow spectra (with widths determined by losses), the trapped modes of the flying trap have spectra with width *Δω*, inversely proportional to the trap duration, *Δω* ~ 1/*τ*_*s*_, all located at the same central frequency *ω*_*t*_ (see Fig. 3a, b), but with different wavevectors *κ*_*n*_^2,3^.

The linearized evolution equation for a weak probe (classical) wavepacket *Ψ*(*z*, *t*) under the action of dispersion $\hat{\beta }=-\frac{{\beta }_{2}}{2}{\partial }_{tt}-i\frac{{\beta }_{3}}{6}{\partial }_{ttt}+\cdots \,$ and the potential *V*(*t*), created by an optical soliton in the frame of reference propagating with the potential (see "Methods" for more details):

1 $$i{\partial }_{z}\Psi (z,t)=-\hat{\beta }\Psi (z,t)+V(t)\Psi (z,t).$$

As long as only *β*_2_ is nonzero in $\hat{\beta }$, this equation is equivalent to the Schrödinger equation for a nonzero-mass particle in potential *V*, with only an exchange *t* ↔ *z*. Thus, the mode structure is the same as in the case of the standard Schrödinger equation, only with the replacement *ω* ↔ *β*. A more detailed consideration, including a discussion of the influence of further effects, such as self-steepening, is given in “Methods”.

Importantly, as further shown in “Methods”, Eq. (1) is valid not only for weak classical wavepackets but also for single-photon Fock states, forming the state $\left\vert \Psi (z,t)\right\rangle=\int\,\Psi (z,t){e}^{-i\omega t}{a}_{\omega }^{{\dagger} }d\omega \left\vert 0\right\rangle$, where ${a}_{\omega }^{{\dagger} }$ is the creation operator for frequency $\omega,\left\vert \left\vert 0\right\rangle \right\rangle$ is the vacuum state. Namely, *Ψ*(*z*, *t*), entering the definition of $\left\vert \Psi (z,t)\right\rangle$ is determined, with good precision, by Eq. (1) for all *z*. This establishes a remarkable duality: all the results discussed here apply equally to weak classical wavepackets and single-photon Fock state wavepackets.

This observation shifts the focus from considering operators to photonic waveshapes^36,37^; the aforementioned duality might be considered as a rather expectable consequence of the quantum-classical correspondence, provided that noise and decoherence are negligible. Yet it provides in our effectively linear situation an extremely powerful tool to analyze genuinely quantum states very directly, a tool which, to the best of our knowledge, has remained unused in the present context. Let us note that, to be able to trap a single photon in a useful way, the trap must be well separated from the trapped state in frequency—that is, the spectral overlap of the trap and the trapped state, although it is formally never zero, must still contain significantly less than one photon.

Whereas the losses through the walls can be exactly zero in a time-trap, the trapped radiation still suffers from other types of losses, such as material absorption and scattering on inhomogeneities. Modern waveguides can have losses of around 0.1 dB/km ^38–40^, and even 0.01dB/km ^41^, resulting in propagation distances of 10–100 km and corresponding trapping lifetimes of *τ*_life_ ~ 30–300 μs (see “Methods” for more discussion). With these losses taken into account, our time-trap can thus demonstrate an effective quality factor *Q* = *ω*_*t*_*τ*_life_/2 ~ 10^9^–10^11^, showing potential to exceed, by orders of magnitude, the performance of the best stationary cavities. This rather long storage time can qualify as a quantum memory ^34,42,43^. The platform suitable for large-*Q* trapping should not only exhibit low material and scattering losses, but also possess a negligible Raman effect, since the latter leads to a frequency shift of the soliton during propagation. The dynamics we consider below occur on much shorter, millimeter to centimeter propagation lengths, so the above-mentioned losses can be safely neglected.

If the trapped state is almost fully decoupled from the external world, how can it be excited? One possibility is to send it directly to the entrance of the waveguide, pre-aligned with the soliton. As soon as the waveforms are pre-selected correctly, after entering the waveguide, the two pulses propagate as the trap and the trapped state, respectively.

**Fig. 4:**
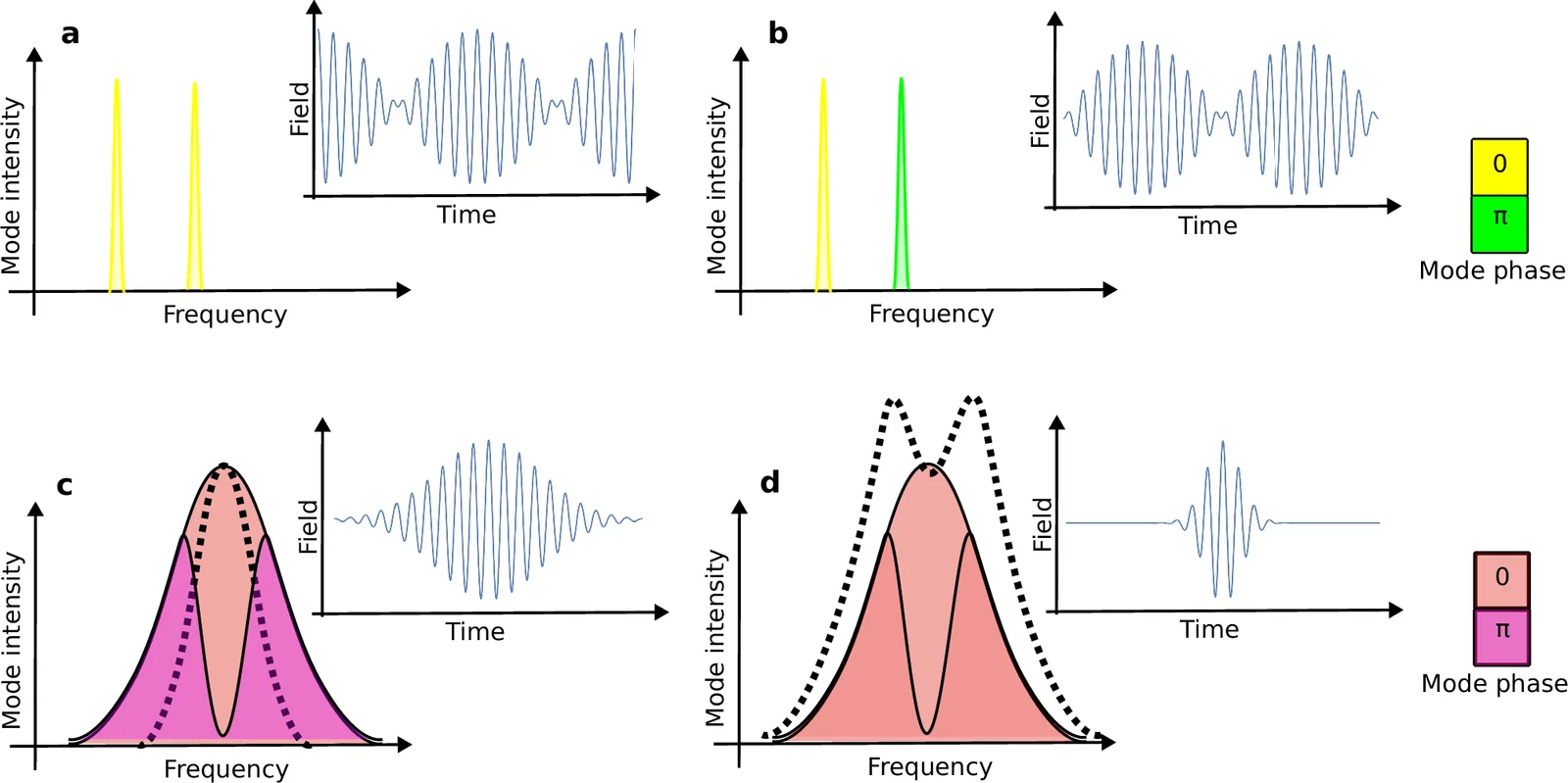
Artistic illustration of beatings between modes in normal and time-trapped cavities. **a**, **b** Beatings between two modes (solid lines) in conventional traps ﻿(cf. Fig. 1b): changes in the relative phases of the modes alter the temporal waveform (insets) but leave the spectral intensity unchanged. **c**, **d** In time-trapping, different temporal modes (solid lines) share the same central frequency (cf. Fig. 3b). Their overlapping spectral components can interfere destructively (**c**) or constructively (**d**). These beatings modify both the resulting spectral intensity (dashed line) and the pulse shape in time (insets).

To demonstrate unusual features of time traps, we start from the standard inter-mode beatings. In Fig. 3c–g, the modes *n* = 0, *n* = 2 with equal amplitudes are simultaneously excited. The simulations here and below use the full nonlinear equation (Eq. (5) in “Methods”). Because these modes have different propagation constants *κ*_0_ ≠ *κ*_1_, the *z*-dependent phase shift ${e}^{iz({\kappa }_{0}-{\kappa }_{1})}$ arises, leading to effectively linear beatings (see also “Methods”). Unlike the beatings in all known stationary cavities, the beatings arising here result in modifications of the pulse shapes – not only in time, but also in frequency—because all modes share the same central frequency *ω*_*t*_ and thus different frequency components can interfere (see Fig. 4). Particularly remarkable states are shown in Fig. 3d–g, demonstrating compression of a pulse with an initial FWHM duration of 28 fs to 4.7 fs during usual linear beatings. Note that both the initial and compressed pulses are Fourier-limited, i.e., they have constant spectral phase (with only jumps by *π*, representing changes in the field sign). Such compression is actually impossible with standard passive linear optics. More generally, beatings allow the transformation of temporal shapes, and this ability increases with the number of modes.

According to the consideration above, the same dynamics arise if single-photon Fock states, rather than weak classical light, are considered. For the trapped photonic wavepackets, we calculate the Wigner function (see “Methods” for details) Fig. 3e, g, which demonstrates negativity, showing that the genuinely quantum nature of the states persists during the dynamics. Note that for a weak classical (coherent) state, the Wigner function is always positive (see “Methods”). Note also that the states in this situation are of the form $\alpha \left\vert {1}_{2}\right\rangle \left\vert {0}_{1}\right\rangle+\beta \left\vert {1}_{0}\right\rangle \left\vert {0}_{2}\right\rangle$ (for some coefficients *α* and *β*, with $\left\vert {i}_{j}\right\rangle$ being the Fock state with *i* photons in time-mode *j*), indicating thereby an entanglement of the same type as is obtained on a beam splitter by splitting a single photon, here, however, for time-modes instead of spatial channens^36,37^.

**Fig. 5:**
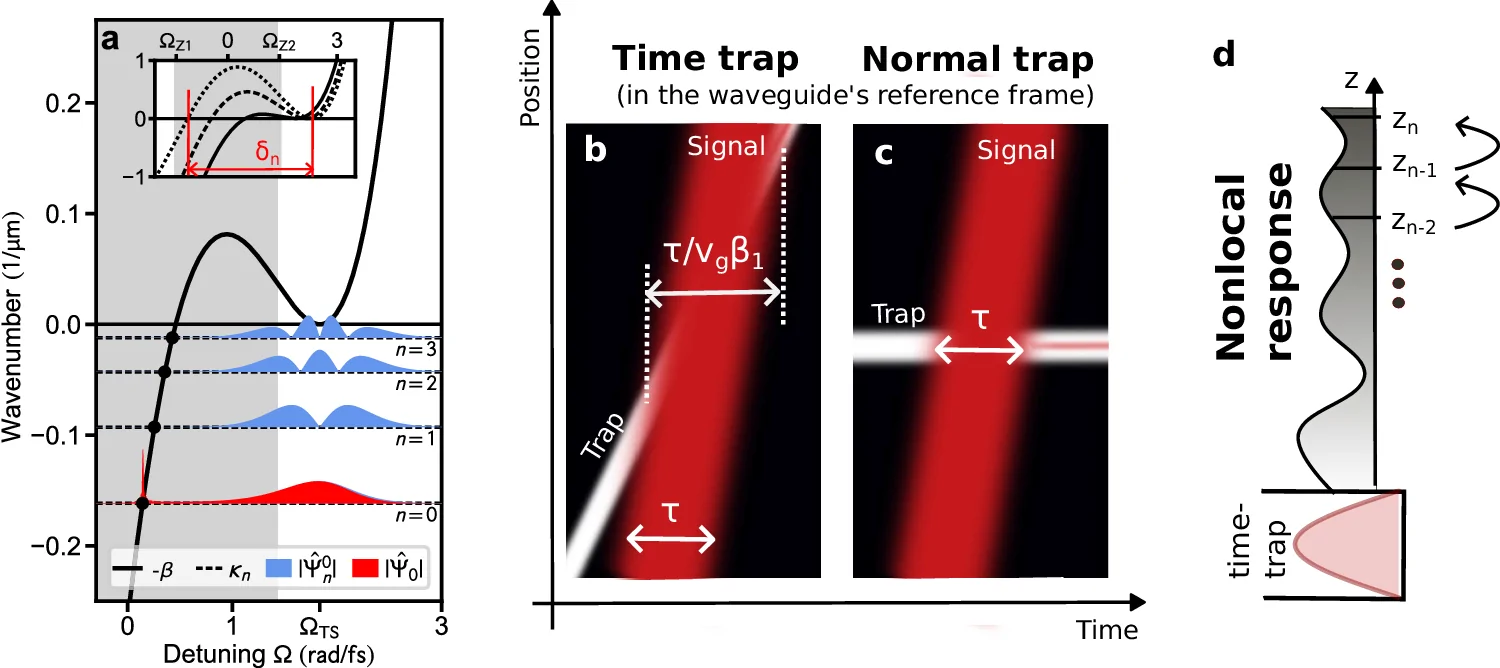
Loading time-trapped states using free-running dispersive waves. **a** Trapped modes in the frequency domain—the fundamental (red) and higher-order (blue) waveforms—are resonantly coupled to dispersive waves (cf. Fig. 2h) in the presence of higher-order dispersion. Black line denotes the dispersion −*β*(*ω*) (in the reference frame of the trap). All trapped modes share the same central frequency but couple to dispersive waves at different frequencies. ${\hat{\Psi }}_{n}^{0}(\Omega )$ and ${\hat{\Psi }}_{0}(\Omega )$ denote the eigensolutions in frequency space for the quadratic and full dispersion, respectively. The inset shows that both the dispersion curve and the resonance positions change when the trap frequency is varied: solid, dashed, and dotted lines correspond to such slightly different frequencies, corresponding to the trapped-state positions at *Ω*_*T*_ = 1.83, 2.14, and 2.30 rad/fs, respectively. The coupling strength *μ*_*n*_ is exponentially sensitive to the frequency-space distance *δ*_*n*_ between the trapped state and the resonant dispersive wave. **b**, **c** Schematics comparing loading geometries for stationary and moving cavities (time-traps) in the waveguide reference frame, highlighting the geometrical factor *v*_*g*_*β*_1_ that increases the “effective pump duration” in the moving case. **d** Illustration of the nonlocal response governed by Eq. (2): nonlocality introduces an effective memory effect, whereby radiation from the cavity at a fixed position *z*_*n*_ depends on all previous positions *z*_*n*−1_, *z*_*n*−2_, ….

**Fig. 6:**
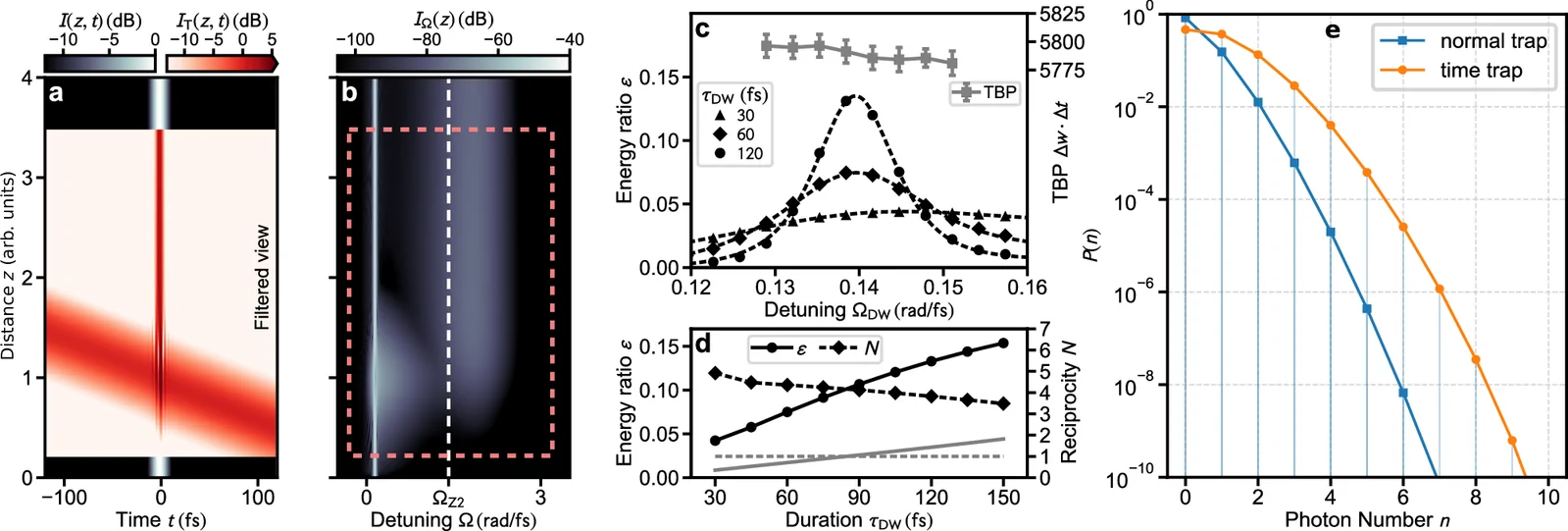
Dynamics of loading of a time-trap in classical and quantum regimes. **a**, **b** Examples of time-trap loading in time and frequency space for the parameters given in Fig. 5a and in the text. The plotting conventions are the same as in Fig. 3; *I*_*Ω*_(*z*) represents the corresponding spectral density. **c**, **d** Dependence of the loaded energy fraction *ϵ* on the detuning *Ω*_DW_ of the dispersive wave pulse (**c**) and on its duration *τ*_DW_ (**d**). In **c**, the left axis shows the corresponding time-bandwidth product; in **d**, it shows the effective reciprocity factor *N*. In **d**, the black curves indicate the dependence for the time-cavity presented here, whereas gray lines show the maximally achievable values for the traditional reciprocal cavity assuming the same decay rate. In **e**, the photon statistics *P*(*n*) are shown for the time-trap with parameters in (**a**) and for the comparable normal trap (*N* = 1) in the situation where single-photon wavepackets are sent repeatedly, leading to the probabilistic accumulation of photons inside the trap.

Furthermore, in the presence of higher-order dispersion, an additional radiation channel is opened, connecting the *n*-th trapped state to resonant radiation at the frequency *ω*_*n*_, determined by the condition *β*(*ω*_*n*_) = − *κ*_*n*_ (see Fig. 5a but also Fig. 2h and^44^). Note that this process can also be considered a form of frequency conversion (*ω*_*n*_ ↔ *ω*_*t*_; Cf. also Fig. 6a, b), working for arbitrarily weak waves, in contrast to most other frequency conversion methods. Also, quite unusually for typical frequency conversion processes, the single initial frequency *ω*_*t*_ is coupled in our case to several output frequencies *ω*_*n*_ for different *n*. Which frequencies are excited is determined by the particular temporal waveform *Ψ*_*n*_(*t*); that is, we have a quite unusual waveform-controlled frequency conversion here.

The coupling strength *μ*_*n*_ between the *n*-th trapped state and the corresponding resonant wave is governed by their overlap integral (see “Methods”), and is exponentially sensitive to the frequency distance *δ*_*n*_ = *ω*_*t*_ − *ω*_*n*_ and to the trapped state duration *τ*_*n*_ (see Fig. 2h and inset to Fig. 5a). The former can be easily controlled by modifying *ω*_*s*_, allowing—together with the pulse duration—control over the strength of this channel.

As soon as the channel is open, energy leakage from the trapped state takes place, leading to decay of the trapped amplitude with a rate *Γ* = 1/*Δz*, *Γ* ∝ ∣μ_*n*_∣^2^ (see “Methods”). The channel also works in the opposite direction, allowing one to load the trap using an incoming pulse at *ω*_*n*_ (Fig. 6a, b). In the example shown in Fig. 6a, b, the fundamental state *n* = 0 of a soliton with *τ*_*s*_ = 6 fs is loaded using a pulsed resonant wave (*Ω*_DW_ ≈ 0.14 rad/fs) with duration *τ*_DW_ = 60 fs. Here, *ϵ* = 8% of the energy is transferred into the trap.

The time-bandwidth product *M* = *ΔtΔω* (where *Δω* is the spectral width of the mode, *Δt* = *Δz*/*v*_*g*_ is the state’s lifetime and v_g_ is the group velocity of the trap) is, for the case of Fig. 6 *M* ≈ 6 × 10^3^ (see Fig. 6c), much larger than the limit *M* = 2 for the traditional stationary cavities (see below for more details). We note that the time-bandwidth limit does not apply to the trapped state alone, but rather to the combined system of the trapped state and the radiation (and other loss) channels.

To get a more general picture we derive the equation governing the intra-trap amplitude *a*(*z*) of the time-cavity (see “Methods” → Equations for the Amplitudes), assuming for simplicity a single trapped state with the eigenvalue *κ*:

2 $${\partial }_{z}a=-i\kappa a-\int\,\,\Gamma (z-{z}^{{\prime} })a({z}^{{\prime} })d{z}^{{\prime} }+\int\,\,\chi (z,{z}^{{\prime} })p({z}^{{\prime} })d{z}^{{\prime} },$$

where *p*(*z*) describes the incident pulse, and $\chi (z,{z}^{{\prime} })$ and *Γ*(*z*) describe the coupling for in- and out-coupled waves, respectively. This equation is noticeably different from the typical known equation describing stationary cavities^31^:

3 $${\partial }_{t}a(t)=-i\kappa a(t)-\Gamma a(t)+\chi p(t),$$

not only because of the replacement *z* ↔ *t*, but also because the in- and out-couplings are integral operators, rather than scalars (*Γ*, *χ*).

In particular, returning to the time-bandwidth product limit, in traditional cavities described by Eq. (3), it arises unavoidably because the decay constant *Γ* defines both the lifetime *Δt* = 1/*Γ* and the linewidth *Δω* = 2*Γ* simultaneously (see “Methods” → Time-Bandwidth Limit for detailed definitions). In our case, this strict relation is broken not only because *Γ* in Eq. (2) is no longer a scalar, but to a much higher degree because *Γ* in Eq. (2) no longer automatically defines the spectral width *Δω*; the latter is now defined by the temporal width of the trap: *Δω* ∝ 1/*τ*_*s*_. That is, *Δt* and *Δω* are now completely decoupled. This allows us, by keeping the pulse short (large *Δω*), to simultaneously have a long interaction time (large *Δt*), which is impossible with the standard time-bandwidth limitation and is promising for enhancing nonlinear interactions^10,45^.

**Fig. 7:**
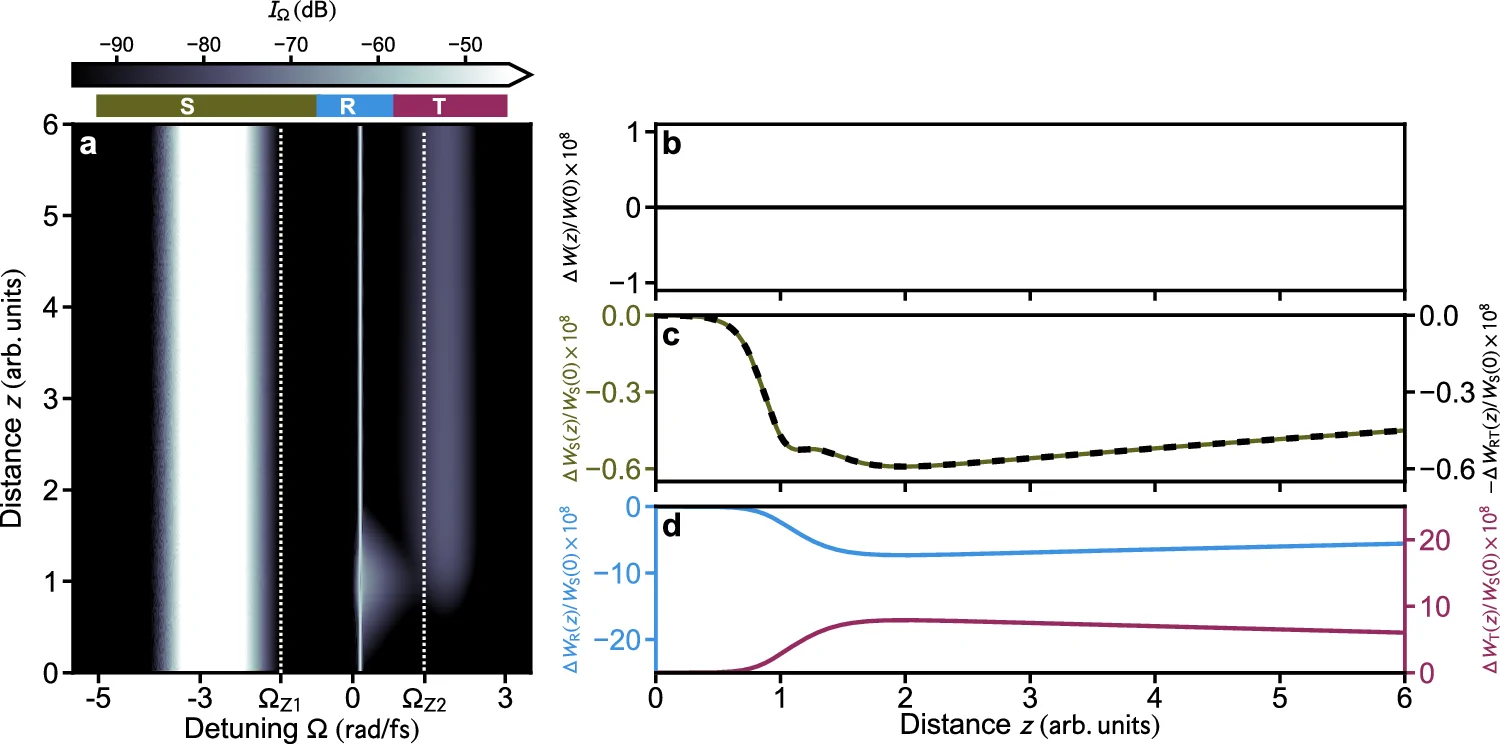
Energy conservation during interaction with a dispersive wave, as shown in Fig. 6a, b. **a** Extended view of the collision region between the dispersive wave and the soliton in the frequency range. **b** Total energy change *ΔW* of the full field as a function of propagation distance *z,* normalized to the propagation before collision *z*_coll_. **c** Energy changes *ΔW*_*S*_ in the soliton energy *W*_*S*_ (solid line) and in the combined reflected and transmitted energy *W*_*RT*_. **d** Separate contributions of the reflected and transmitted waves. Note the scale of modification, on the order of 10^−8^ relative to the soliton energy. See “Methods” → Model and Parameters for the definition of the corresponding energies.

Furthermore, we observe that time cavities are able to load energy from external pulses fundamentally more efficiently than normal cavities. Indeed, integrating Eq. (3), one can easily obtain (see “Methods” for more details) that the maximal fraction of energy that can be loaded from a pulse of finite duration *τ* into the cavity is:

4 $$\epsilon=2NF\frac{\tau }{\Delta t},$$

where *F* is a pulse-dependent form factor (*F* = *π*^2^/2 for a sech-shaped pulse) and *N* = *χ*^2^/2*Γ*. The well-known^31^ reciprocity condition *N* = 1 determines the ratio between the in- and out-couplings and follows from Eq. (3), dictated by time-reversibility and energy conservation. In particular, nonreciprocal cavities with *N* > 1, which can be created by breaking time symmetry via external magnetic fields or rotation, allow, according to Eq. (4), storage of a larger fraction of the input pulse than standard reciprocal cavities. Even though the dynamics in our case are formally fully reversible and energy conserving (see Fig. 7; also “Methods” and Sec. III of Supplementary, in particular Supplementary Figs. 1 and 2), we observe a similar effect: we can store more energy in the trap than the maximal value prescribed by Eq. (4) under the assumption *N* = 1. This effect–for reasons that will become clear below–can be described as effective nonreciprocity. We quantify the effective nonreciprocity by expressing *N* in Eq. (4)and using values of *ϵ* and *Δt* obtained either numerically (see Fig. 6d) or analytically. As we see in Fig. 6d, in general, *N* ≠ 1.

Careful consideration (see “Methods”) reveals two different sources of this effect: first, a purely geometrical factor, illustrated in Fig. 5b, c, representing the simple fact that the pump pulse and the trap have nearly equal group velocities. Their interaction time is thereby increased by a factor 1/*v*_*g*_*β*_1_ compared to a stationary pulse. This leads to exactly the same effect as increasing *τ* by the same factor in Eq. (4) (see “Methods” → Limit on Energy Loading from Dispersive Wave to the Trapped State for more details). However, since *τ* is fixed, we instead include the factor 1/*v*_*g*_*β*_1_ in *N*, denoted as *N*_geom_ = 1/*v*_*g*_*β*_1_. For the parameters of Fig. 6a and *v*_*g*_ = 0.3 μm/fs, we have *N*_geom_ ≈ 3.7.

Second, as previously noted, the coupling constants in Eq. (2) are no longer scalars. This nonlocality results in a memory effect (see Fig. 5d), leading to another contribution to *N*, denoted *N*_nonlocal_, which, for the above-mentioned parameters, is *N*_nonlocal_ ≈ 1.18, noticeably smaller than *N*_geom_.

The resulting effective nonreciprocity is the product of these two factors: *N* = *N*_nonlocal_*N*_geom_. This full effective nonreciprocity is shown in Fig. 6d as a function of the input pulse duration, assuming *v*_*g*_ mentioned above. Note that the effective nonreciprocity, while enabling more efficient loading of the cavity, is free from the disadvantages of common nonreciprocal systems, namely their fundamentally non-conservative character, meaning that nonreciprocity inevitably introduces additional noise^46^. In contrast, the dynamics in our case remain fully conservative, so that no additional noise is introduced (cf. Fig. 7, where full energy conservation is shown).

From Eq. (4), it follows that the cavity enhancement factor (if we define it as the ratio of the intensities inside and outside the cavity) is larger by the same factor *N* in the time-cavity than in a traditional cavity of the same spatial size and decay rate. Moreover, as mentioned above, time-cavities can combine an ultrashort size with low decay rates, so that an even larger enhancement-factor excess can be expected.

All this promises straightforwardly an effective memory for photons. Indeed, if we load our trap with the single photon Fock state $\left\vert 1\right\rangle$ in the situation described by Figs. 5 and 6, the state created inside the trap is obviously $\approx \sqrt{\epsilon }\left\vert 1\right\rangle$. If we let the remaining photonic wavepacket pass through the trap again and again (with the right phase), after ${N}_{p}\approx 1/\sqrt{\epsilon }$ passages we store the whole photon, assuming that the escape rate is negligible, that is, *τN*_*p*_ ≪ *Δt* (in this estimation, neglect the pulse-to-pulse separation for simplicity). According to Eq. (4), this is achieved for $N/\sqrt{\epsilon }\gg 1/2F\approx 1/10$, which is satisfied for Fig. 6c, d, where this ratio varies between 75 and 220 (whereas for normal cavities its typical value is of the order of 10).

Alternatively, one can consider a similar “repeating impact” situation, but now with single-photon Fock states in every incoming pulse (meaning that, say, *m* pulses contain exactly *m* photons). In this case, again, every time a part $\approx \sqrt{\epsilon }\left\vert 1\right\rangle$ of the photon is stored. Now, however, a multiphoton state is created in the trap: after the *m*th pulse we have $\approx {(\left\vert 0\right\rangle+\sqrt{\epsilon }\left\vert 1\right\rangle )}^{m}$ (again, assuming the losses are neglected for simplicity). This state can be described by a binomial photon-number statistics *P*(*n*). The effective nonreciprocity gives a noticeable advantage also in this situation: the higher values of *n* are excited with higher probability than is possible to achieve with traditional reciprocal cavities. An example is given in Fig. 6e, where the exemplary statistics *P*(*n*) for a time-trap and for a comparable normal reciprocal trap (meaning *N* = 1 in Eq. (4)) is shown for *m* = 10.

**Fig. 8:**
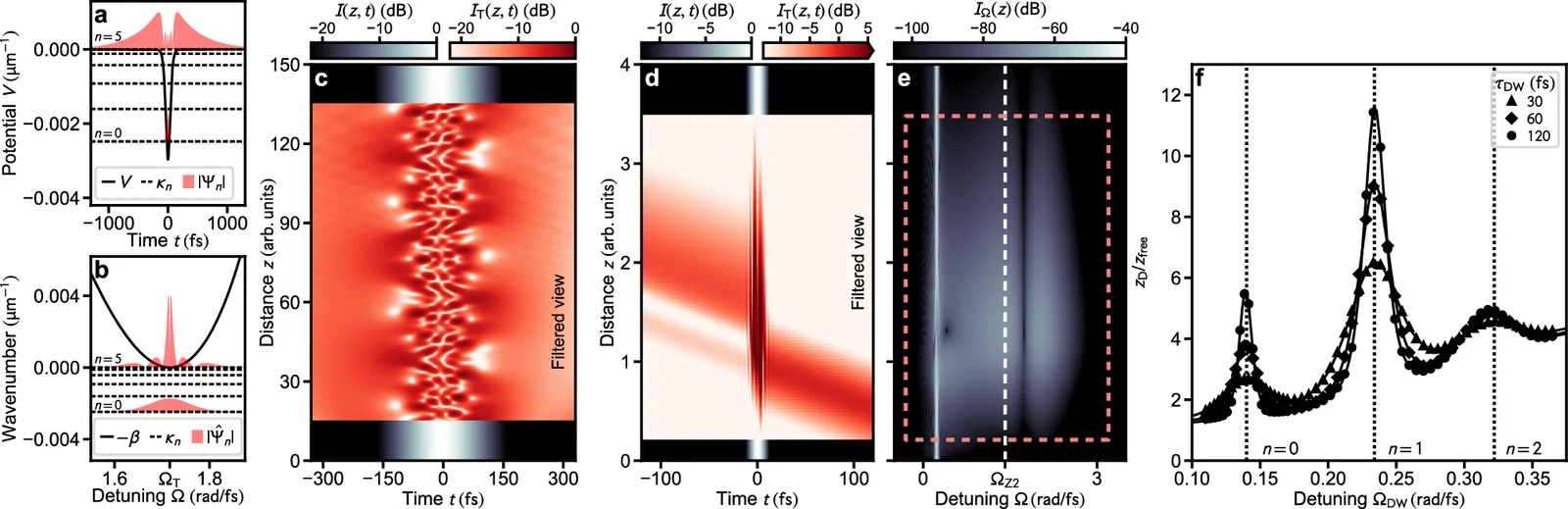
Halo states and anomalous transmission delays. **a**, **b** Halo state corresponding to mode *n* = 5, shown in the time domain (**a**) and the frequency domain (**b**). **c** Temporal mode beatings when all modes *n* = 0 to *n* = 5 are excited, including the halo state. **d**, **e** Excitation of the *n* = 1 trapped state results in anomalously large transmission delays, shown in the time domain (**d**) and frequency domain (**e**). **f** Propagation delay *z*_*D*_ as a function of the detuning *Ω*_DW_ of the input pulse. The delay *z*_*D*_ is normalized to *z*_free_, which is the delay observed in the absence of the potential. Vertical lines indicate the resonant frequencies *ω*_*n*_ corresponding to the *n*-th trapped states. The plotting conventions the same as in the previous figures.

As mentioned above, time-trapping in several respects more closely resembles the trapping of massive particles than that of massless photons. One of the most exotic trapped states for massive particles is known as a halo state ^47,48^. These appear in short-range potentials but extend far beyond the potential region (see “Methods” for more details). Halo states have so far been observed in nuclear physics ^47^, cold gases^49^, and in molecular physics^50,51^ for single-, double-, and three-nuclear systems, but have never been predicted or observed in optics. To obtain a halo state, one needs an eigenvalue *κ*_*n*_ very close to zero in a short-range potential, while still remaining negative. This condition can also be realized in our system. An exemplary resulting halo state is shown in Fig. 8a, b. Furthermore, Fig. 8c shows exemplary beatings involving halo states.

Last but not least, we also observe huge anomalous delays in transmission, as shown in Fig. 8d–f, where the input pulse is near resonance with the state *n* = 1. The delay for adjacent resonances is noticeably less pronounced (Fig. 8f). In this case, the input pulse is almost fully loaded into the trapped state and then gradually re-emitted, thereby dwelling in the vicinity of the cavity. This large group delay is likely due to destructive interference between the trapped state and the passing dispersive wave.

The fact that our trap, unlike conventional traps, is not constrained by the time-bandwidth limit and demonstrates effective nonreciprocity, introduces a new dimension to the longstanding and actively debated^52–55^ question of delay times experienced by quantum particles of any physical nature when traversing a potential. Let us note once again that in our case the corresponding dynamics—as with all dynamics considered in this article—are equally valid for arbitrarily weak classical and single-photon quantum wavepackets.

## Discussion

As we have shown, time-trapping exhibits highly unusual properties. On the one side, the interaction of the strong trap with the weak trapped pulse can be formally described using the language common to nonlinear interactions as the effect of cross-phase modulation. The alternative, extremely fruitful framework is to consider this as an effectively linear process in time-optics. Indeed, such trapping is described by a linear, Schrödinger-like equation with *z* ↔ *t*. Yet, this equation has fundamental differences from the equations describing traditional passive optical trapping. In certain aspects, time-trapping resembles more the trapping of massive particles rather than massless photons. In particular, it can exhibit formally zero leakage through the walls—in the same way as it happens in traps for, for instance, electrons, in contrast to traditional traps for photons. Because of this, time-traps enable the effective photon trapping on an extremely broad petahertz-scale bandwidth while promising quality factors as high as *Q* ~ 10^9^-10^11^.

Coupling to dispersive waves at different frequencies can be enabled in a tunable manner, allowing one to “load” and “unload” time-traps. This coupling breaks several limits inherent to traditional optics: first, time-traps can be loaded faster than fundamentally possible for traditional traps, mimicking in this respect nonreciprocal cavities, but in a fully conservative way. The effect arises from the unusual geometric nature of time traps, flying at the speed of light and thereby enhancing the interaction distance with the incoming wave, but also due to the nonlocality inherent to the time-trapping dynamics. Second, the time-bandwidth limit—the inherent coupling between the coupling (decay time) of a single trapped mode and its bandwidth, hard-coded into the traditional Lorentzian lineshapes in optics (and physics in general)—is not valid anymore for time-traps. Even if the breaking of the time-bandwidth limit in time-traps may appear somewhat trivial, taking into account the intrinsically broadband nature of time-trapped modes, it clearly highlights how different the physics of time-traps is compared to that of conventional traps. It furthermore holds strong promise for surpassing the limits of nonlinear interactions in traditional optics^45^.

Time-trapped modes are genuine single-mode states with a well-defined phase and wavevector, even if they have a petahertz-scale linewidth; all modes share the same central frequency. These mathematically obvious, but puzzling for traditional passive linear optics, properties of time-trapped states allow for unusual optics. For instance, the latter fact can be used to reshape pulses both in the frequency and time domains via trivial linear mode beating; the time-trapping process itself can thereby alternatively be viewed as a waveshape-sensitive frequency conversion. More generally, ultrafast reshaping, frequency conversion, exotic halo states, and anomalously large yet tunable transmission delays, demonstrated here, offer a new paradigm for controlling photonic wavepackets and temporal modes^36,37^ on the few-cycle scale.

Being conservative and effectively linear, this framework is fully applicable to arbitrarily weak classical and quantum states of light—including single photons—in contrast to conventional approaches ^56^, and thereby holds particular promise for applications in ultrafast quantum photonics^10^, in particular for effective memories, frequency conversion, waveshape reshaping for ultrafast photon wavepackets, and creation of unconventional multiphoton states of light.

## Methods

### Model and parameters

We model *z*-propagation of solitary wave cavities and their trapped states in terms of a complex-valued field *A*(*z*, *t*) using the modified nonlinear Schrödinger equation (NSE):

5 $$\begin{array}{r}i{\partial }_{z}{A}_{\omega }=-\beta (\omega ){A}_{\omega }-\gamma (\omega ){\left(| A{| }^{2}A\right)}_{\omega },\end{array}$$

where ${A}_{\omega }={A}_{\omega }(z)={{\mathcal{F}}}[A(z,t)]$ is the Fourier transform ${{\mathcal{F}}}$ (from the time to the frequency domain) of *A*(*z*, *t*), *β*(*ω*) = (*β*_2_/2)*ω*^2^ + (*β*_4_/24)*ω*^4^ is the propagation constant, and *γ*(*ω*) = *γ*_0_ + *γ*_1_*ω* is the nonlinear coefficient, accounting for self-steepening (represented by the linear dependence on frequency). For the simulations in the main article, we used the parameters *β*_2_ = 1 fs^2^/μm, *β*_4_ = − 1 fs^4^/μm, *γ*_0_ = 1 W^−1^/km, and *γ*_1_ = 0.05 W^−1^fs/km.

Although, for the sake of simplicity and generality, we do not consider any specific waveguide, these parameters are chosen such that the nonlinear and dispersion lengths correspond to those of typical SiO_2_ waveguides. We deliberately keep our theory as simple as possible by neglecting some effects that might appear in specific waveguides, such as the Raman effect, free-electron generation, two-photon absorption, multimode propagation, and so on.

To obtain Eq. (1) we linearize Eq. (5) in the vicinity of a fundamental nonlinear Schrödigner soliton $u(t)=\sqrt{{P}_{0}}\,{\mbox{sech}}\,(t/{\tau }_{s}){e}^{-i{\omega }_{s}t}$, centered at *ω*_*s*_ with peak intensity ${P}_{0}=| {\beta }_{2}({\omega }_{s})| /[\gamma ({\omega }_{s}){\tau }_{s}^{2}]$ by taking *A*(*z*, *t*) = *u*(*z*, *t*) + *Ψ*(*z*, *t*) and transforming the frame of reference to match the group velocity *v*_*g*_ of the soliton. Also, in Eq. (1), we neglect self-steepening for clarity. We discuss the impact of self-steepening on the trapped states in the section Trapped States below.

Even though Eq. (1) provides an equivalent way to Eq. (5) to describe the dynamics of low-intensity probe waves (as we verified numerically), we used Eq. (5) for the simulations in the main article for self-consistency.

To monitor the conservation of energy in Fig. 7 when performing pulse propagation simulations using Eq. (5), we consider the total energy

6 $$W(z)=\int\,| {A}_{\Omega }(z){| }^{2}\,{{\rm{d}}}\Omega .$$

To monitor a transfer of energy between different parts of the spectrum, we distinguish the following frequency regions: a region *X*_S_ = (−5, −0.7) rad/fs, which holds the soliton, *X*_R_ = (−0.7, 0.8) rad/fs, which holds the dispersive wave and the radiation emanating from the trapped state, and, *X*_T_ = (0.8, 3) rad/fs, holding the trapped state. The corresponding energies read ${W}_{{{\rm{S}}}}(z)=\int_{{X}_{{{\rm{S}}}}}| {A}_{\Omega }(z){| }^{2}\,{{\rm{d}}}\Omega$, ${W}_{{{\rm{R}}}}(z)=\int_{{X}_{{{\rm{R}}}}}| {A}_{\Omega }(z){| }^{2}\,{{\rm{d}}}\Omega$, and ${W}_{{{\rm{T}}}}(z)=\int_{{X}_{{{\rm{T}}}}}| {A}_{\Omega }(z){| }^{2}\,{{\rm{d}}}\Omega$.

The energy in the combined range *X*_R_ ∪ *X*_T_ is ${W}_{{{\rm{RT}}}}(z)=\int_{{X}_{{{\rm{R}}}}\cup {X}_{{{\rm{T}}}}}| {A}_{\Omega }(z){| }^{2}\,{{\rm{d}}}\Omega$.

### Analogy to the dynamics of a single-particle wavefunction

Here we emphasize the analogy to standard quantum mechanics. Indeed, Eq. (1) can be seen as a one-to-one mapping of the one-dimensional single-particle Schrödinger equation for the wavefunction *Ψ*(*x*, *t*), describing *x*-dependent wavefunctions *Ψ* evolving in time *t*:

7 $$i\hslash {\partial }_{t}\Psi (x,t)=\hat{H}\Psi (x,t),$$

where *ℏ* is the reduced Planck constant and $\hat{H}$ is the Hamiltonian. Very often, $\hat{H}$ has the form $\hat{H}={\hat{H}}_{0}+V(x)$, consisting of the free-particle Hamiltonian ${\hat{H}}_{0}=-({\hslash }^{2}/2m){\partial }_{xx}$ and the potential *V*(*x*).

To obtain Eq. (1) from Eq. (7), we make the substitutions *ℏ* → 1, *t* → *z*, *x* → *t*, *Ψ*(*x*, *t*) → *Ψ*(*t*, *z*), and $\hat{H}\to -\hat{\beta }+V(t)$. In particular, all the dynamics in Eq. (1) are fully unitary and energy-conserving. Later on, we will use terminology and methods inspired by this analogy. As discussed below, Eq. (1) is also a valid equation describing a single-photon wavepacket.

### Trapped States

When both the soliton and the weak pulse have narrow spectra (higher-order dispersion can be neglected), we have (cf. previous sub-section):

8 $$\hat{H}\approx {\hat{H}}^{(0)}=\frac{{\beta }_{2}({\omega }_{t})}{2}{\partial }_{tt}+V(t)$$

with

9 $$V(t)=-2\gamma ({\omega }_{t})| u(t){| }^{2},$$

where *u*(*t*) is the soliton waveform (see Sec. “Model and Parameters”) and *ω*_*t*_ is the frequency of the trapped state. Introducing the parameter *ν* as

10 $$\nu=-\frac{1}{2}+{\left[\frac{1}{4}+4\frac{\gamma ({\omega }_{s})}{\gamma ({\omega }_{t})}\frac{{\beta }_{2}({\omega }_{t})}{{\beta }_{2}({\omega }_{s})}\right]}^{1/2},$$

the potential can be written as

11 $$V(t)=\frac{{\beta }_{2}({\omega }_{t})}{2}\frac{\nu (\nu+1)}{{\tau }_{s}^{2}}{{\mbox{sech}}}^{2}\left(\frac{t}{{\tau }_{s}}\right).$$

The formal analogy mentioned in the previous section allows us to identify the resulting equation as a Schrödinger equation for a fictitious particle of mass *m* = -1/*β*_2_(*ω*_*t*_), moving under the influence of potential *V*(*t*). Trapped states have the form $\psi (z,t)={\psi }_{n}(t){e}^{-i{\kappa }_{n}z}$ with real-valued eigenvalues *κ*_*n*_ < 0 for a finite number of modes $n=0,1,\ldots,{N}_{\max }-1$, where ${N}_{\max }=\lfloor \nu \rfloor+1$. The wavenumber eigenvalues are given by^2,3,57,58^:

12 $${\kappa }_{n}=\frac{{\beta }_{2}({\omega }_{t})}{2{\tau }_{s}^{2}}{(\nu -n)}^{2},$$

and the corresponding (not normalized) wavefunctions are

13 $${\psi }_{n}(t){=}_{2}{F}_{1}\left[{a}_{n},{b}_{n};\frac{1}{2};x(t)\right]y(t)\quad \,{{\rm{for}}} \,{{\rm{even}}}\,n,$$

14 $${\psi }_{n}(t){=}_{2}{F}_{1}\left[{a}_{n}+\frac{1}{2},{b}_{n}+\frac{1}{2};\frac{3}{2};x(t)\right]y(t){y}^{{\prime} }(t)\quad \,{{\mbox{for}}} \,{{\rm{odd}}}\,n,$$

where

15 $$y(t)={\cosh }^{\nu+1}(t/{\tau }_{s}),\quad {y}^{{\prime} }(t)=\sinh (t/{\tau }_{s}),\quad x(t)=-{y}^{{\prime} 2}(t),$$

16 $${a}_{n}=\frac{1}{2}(\nu+1-\sqrt{| {\kappa }_{n}| }{\tau }_{s}),\quad {b}_{n}=\frac{1}{2}(\nu+1+\sqrt{| {\kappa }_{n}| }{\tau }_{s}),$$

and _2_*F*_1_[⋅] is the Gaussian hypergeometric function^59^. In particular, the fundamental solution (*n* = 0) is

17 $${\psi }_{0}(t)=\frac{1}{{\cosh }^{\nu }(t/{\tau }_{s})}.$$

Since *κ*_*n*_ are fully real, this indicates the absence of intrinsic losses caused by the potential walls—that is, zero leakage through the walls. For a detailed discussion of how this behavior differs from that predicted by Fresnel laws, see the main text. As soon as the potential is attractive, at least one trapped state exists.

In the presence of higher order dispersion, the waveshapes are deformed. However, in the typical situation, at least for the fundamental state, this deformation is rather small. For instnance, this is the case in Fig. 5a (observe the (extremely small) difference between ${\hat{\Psi }}_{0}$ and ${\hat{\Psi }}_{0}^{(0)}$).

Modes can exhibit beatings, as discussed in the article, if the initial condition is a superposition of multiple modes:

18 $$\Psi (t,z=0)={\sum}_{n}{a}_{n}{\psi }_{n}(t).$$

Upon propagation, this waveform evolves as

19 $$\Psi (t,z)={\sum}_{n}{a}_{n}{\psi }_{n}(t){e}^{-i{k}_{n}t}.$$

As discussed in the article, both the pulse duration and the spectral width evolve during such beatings—a unique feature compared to standard optics (see also Fig. 4).

### Halo states

A halo state is typically defined as a bound state whose spatial extent reaches far beyond the potential. The family of such states includes many examples with different numbers of interacting bodies, such as Efimov states, Borromean states, and others^47,60,61^. In our case, the highest trapped state has  $n={{\mathcal{N}}}$ (where ${{\mathcal{N}}}={N}_{\max }-1$, *N*_max_ is the total number of trapped states), as it approaches the continuum ${\kappa }_{{{\mathcal{N}}}}\approx 0$, still remaining negative ${\kappa }_{{{\mathcal{N}}}} < 0$, represents a halo state. Indeed, an asymptotic analysis shows that the tail of such a halo state *ψ*(*t*) decays as

20 $$\psi (t) \sim {e}^{-\sqrt{2| {\kappa }_{{{\mathcal{N}}}}| /| {\beta }_{2}| }| t| },$$

while the trapping potential decays as

21 $$V(t)\propto {e}^{-2| t| /{\tau }_{s}}.$$

Therefore, if

22 $${\tau }_{s}^{2}\ll 2| {\beta }_{2}| /| {\kappa }_{{{\mathcal{N}}}}|,$$

then *ψ*(*t*) extends far outside the soliton trap.

### Single-photon quantum wavepacket propagation

We can easily see that Eq. (1) is a valid description at the single-photon level in our situation. To show this, we use the *z*-propagating master-equation approach recently developed in^62,63^, which describes quantum evolution in arbitrary dispersion profiles as well as in the presence of frequency-dependent nonlinearity. (Although in our case, alternative approaches^62–67^ yield essentially the same result.)

Here we describe only the essential steps; details can be found in the Supplementary (Sec. I). The dynamics in^62,63^ is, under reasonable approximations, described by a stochastic Schrödinger equation (stSE), equivalent to Eq. (5) with the addition of an amplitude-dependent noise term *σ*(*A*_*ω*_) on the right-hand side (the noise is in general state-dependent).

The stochastic solution *A*_*ω*_(*z*), obtained by solving this stSE, represents the quantum density matrix *ρ*(*z*) = ∫*ρ*_*ω*_(*z*)*dω* in the so-called positive *P*-representation^64,65^, in the form of a probability distribution *P*_*ω*_(*A*_*ω*_(*z*), *B*_*ω*_(*z*)), with ${\rho }_{\omega }=\int\,\int\,\,{P}_{\omega }({A}_{\omega },{B}_{\omega })\left\vert {A}_{\omega }\right\rangle \left\langle {B}_{\omega }^{*}\right\vert /\langle {A}_{\omega }| {B}_{\omega }^{*}\rangle {d}^{2}{A}_{\omega }{d}^{2}{B}_{\omega }$ (for each *z* and *ω*).

The corresponding stSE can be linearized similarly to the method used in the previous section, yielding Eq. (1) plus corresponding noise terms. As long as the noise terms can be neglected (see below), we can describe the single-photon wavepacket in the form $\rho (z)=\left\vert \Psi (z)\right\rangle \left\langle \Psi (z)\right\vert$, where $\left\vert \Psi (z)\right\rangle={\sum }_{\omega }{\Psi }_{\omega }(z){a}_{\omega }^{{\dagger} }\left\vert 0\right\rangle$, ${a}_{\omega }^{{\dagger} }$ is the creation operator for a photon at frequency *ω*, and $\left\vert 0\right\rangle$ is the vacuum state. We then obtain exactly Eq. (1) for $\Psi (z,t)={{\mathcal{F}}}[{\Psi }_{\omega }(z)]$. This can be shown using the fact that *Ψ*_*ω*_ may be arbitrarily small, so that the coherent state $\left\vert {A}_{\omega }\right\rangle$ can be well approximated as $\left\vert 0\right\rangle+{\Psi }_{\omega }{\left\vert 1\right\rangle }_{\omega }$, where ${\left\vert 1\right\rangle }_{\omega }$ is the single-photon Fock state at frequency *ω*, allowing us to perfectly identify the equations for *A*_*ω*_ and *Ψ*_*ω*_.

The influence of the noise terms is estimated in detail in the Supplementary (Sec. IV), using the stSE developed in^62,63^. In our particular case, the noise leads to decoherence of the trapped wavepacket. The corresponding decoherence distances are much greater than 1 km for our parameters — that is, entirely negligible for the effects considered here.

### Visualization of the dynamics; spectrogram and wigner-function

To assess and visualize the dynamics of weak classical waves, we first separate it from the trap (soliton) part of the whole field *A*_*Ω*_(*z*) in Eq. (5) [here we use the detuning *Ω* instead of frequency *ω* (cf. caption to Fig. 1)] as $\Psi (z,t)=\int\,{A}_{\Omega }(z)\Theta (\Omega )\exp (-i\Omega t)\,{{\rm{d}}}\Omega$ with Heaviside step function *Θ*. *Ψ*(*z*, *t*) is small enough in comparison to the trap, and, as mentioned before, obeys the linear equation Eq. (1). Linearity makes the absolute amplitude irrelevant; therefore, for convenience, we normalize *Ψ* such that ∫ ∣*Ψ*∣^2^d*t* = 1. We also define the corresponding normalized intensities *I*(*z*, *t*) = ∣*Ψ*(*z*, *t*)∣^2^, as well as the amplitude ${\Psi }_{\Omega }(z)={{\mathcal{F}}}[\Psi (z,t)]$ and intensity *I*_*Ω*_(*z*) = ∣*Ψ*_*Ω*_(*z*)∣^2^ in the frequency space. These functions are plotted in Figs. 3c, 6a, b, 7a, and 8c–e. The joint time-frequency analysis is then performed in terms of the spectrogram^68^ (for some selected *z*)

23 $$P(\Omega,t)=\frac{1}{2\pi }\left| \int\Psi (z,{t}^{{\prime} })h({t}^{{\prime} }-t){e}^{i\Omega {t}^{{\prime} }}{{\rm{d}}}{t}^{{\prime} }\right| ^{2},$$

wherein $h({t}^{{\prime} }-t)=\exp [-{({t}^{{\prime} }-t)}^{2}/2{\sigma }^{2}]$ is a Gaussian window function with root-mean-square width *σ*. For the visualization of the spectrogram (23) in Fig. 3d, f, we considered *σ* = 10 fs and a decibel scale $10{\log }_{10}[P(t,\Omega )/{P}_{0}]$ with reference to the peak-value ${P}_{0}=\max [P(t,\Omega )]$.

As it is mentioned in the article, Eq. (1) can also be considered to describe the single-photon quantum states via the waveshape function *Ψ*(*z*, *t*) (see also the previous section). Thus, we can interpret *Ψ*(*z*, *t*) obtained from Eq. (5) and the filtering described in the previous paragraph as a single-photon waveshape. To access its quantum properties in Fig. 3e, g, we use the Wigner function (for some fixed *z*):

24 $$W(\Omega,t)=\frac{1}{2\pi }\int\,{\Psi }^{*}(z,t-\frac{1}{2}{t}^{{\prime} })\Psi (z,t+\frac{1}{2}{t}^{{\prime} }){e}^{i\Omega {t}^{{\prime} }}\,{{\rm{d}}}{t}^{{\prime} }.$$

This definition was used to plot the Wigner function in Fig. 3e,g. Negative values of *W* indicate the presence of genuinely nonclassical features. We note that this is a single-particle version of the Wigner function. The quantum Wigner function for the classical light (multiphoton coherent state) should be defined more generally as

25 $$W(x,p)=\frac{1}{\pi \hslash }\int _{-\infty }^{\infty }dy\left\langle x-y\right\vert \hat{\rho }\left\vert x+y\right\rangle {e}^{2ipy/\hslash },$$

with

26 $$x=\sqrt{\frac{\hslash }{2}}\left(\hat{a}+{\hat{a}}^{{\dagger} }\right),\qquad p=-i\sqrt{\frac{\hslash }{2}}\left(\hat{a}-{\hat{a}}^{{\dagger} }\right).$$

Introducing $\alpha=\frac{x+ip}{\sqrt{2\hslash }}$, for the classical (coherent) state with amplitude *α*_0_ we obtain

27 $${W}_{{\alpha }_{0}}(\alpha )=\frac{2}{\pi }\exp \left(-2{\left\vert \alpha -{\alpha }_{0}\right\vert }^{2}\right).$$

In this case, *W *≥ 0, indicating the classical nature of the classical (coherent) state.

### Equations for the amplitudes

Here, we derive the equations for the amplitudes of a trapped state coupled to the continuum. We write

28 $$\hat{H}={\hat{H}}_{0}+\hat{W},\quad {\hat{H}}_{0}=\frac{{\beta }_{2}}{2}{\partial }_{tt}+V(t)+(1-U(t))\delta \hat{\beta },\quad \hat{W}=U(t)\delta \hat{\beta },$$

where $\delta \hat{\beta }=-\hat{\beta }-\frac{{\beta }_{2}}{2}{\partial }_{tt}$ (with this definition, $\delta \hat{\beta }$ does not contain the terms with ∂_*t*_ and ∂_*tt*_). The function *U*(*t*) is chosen to be unity near the localization of *V*(*t*) and zero far from it, and is smooth enough in the transition region to avoid generating reflected waves (see Sec. I of Supplementary for details). With this construction, the trapped eigenstates of ${\hat{H}}_{0}$ closely approximate those of $\hat{H}$, while the free states of ${\hat{H}}_{0}$ at ∣*t*∣ → *∞* have the same asymptotic wavevectors as those of $\hat{H}$. The operator $\hat{W}$ facilitates coupling between the eigenstates of ${\hat{H}}_{0}$, which are otherwise orthogonal and uncoupled. We expand our signal as:

29 $$\Psi (t,z)={\sum }_{i}{a}_{i}(z){\Psi }_{i}(t)+{\sum }_{\omega }{a}_{\omega }(z){\Psi }_{\omega }(t),$$

where *Ψ*_*i*_(*t*) and *Ψ*_*ω*_(*t*) are the bound and unbound eigenstates of ${\hat{H}}_{0}$, respectively, and *a*_*i*_(*z*) and *a*_*ω*_(*z*) are the corresponding amplitudes. The continuum eigenstates have plane-wave asymptotics *Ψ*_*ω*_(*t*) ~ *e*^−*iωt*^ as ∣*t*∣ → *∞*. The normalization follows standard conventions^31^: ∣*a*_*i*_∣^2^ gives the energy of the bound mode, while the energy of the continuum radiation is ∫ ∣*a*_*ω*_∣^2^*dω*. 

Substituting this expansion into Eq. (1), using the orthogonality of the eigenstates of ${\hat{H}}_{0}$, and replacing sums with integrals (see Sec. I of Supplementary for details), we obtain:

30 $$i{\partial }_{z}{a}_{i}={\kappa }_{i}{a}_{i}+\int\,{\mu }_{i\omega }{a}_{\omega }d\omega,\,\,i{\partial }_{z}{a}_{\omega }=-{\beta }_{\omega }{a}_{\omega }+{\mu }_{\omega i}{a}_{i},$$

where we for simplicity restricted our consideration by only one trapped state *i*, and

31 $${\mu }_{i\omega }\approx \int\,{\Psi }_{i}^{*}(\omega^{\prime} )\delta \beta (\omega^{\prime} ){\Psi }_{\omega }(\omega^{\prime} ){{\rm{d}}}\omega^{\prime},{\mu }_{\omega i}={\mu }_{i\omega }^{*}$$

are the coupling matrix elements. Here, ${\Psi }_{i}(\omega^{\prime} ),{\Psi }_{\omega }(\omega^{\prime} )$, and $\delta \beta (\omega^{\prime} )$ are the Fourier transforms of *Ψ*_*i*_(*t*), *Ψ*_*ω*_(*t*), and $\hat{\delta }\beta$, respectively. In the main text, we denote these matrix elements as the coupling strengths  ${\mu }_{i}={\mu }_{i{\omega }_{i}}$.

The trapped mode spectrum *Ψ*_*i*_(*ω*) decays exponentially with detuning *δ*_*i*_ = *ω* − *ω*_*t*_ (for large enough ∣*δ*_*i*_∣), and so does the coupling matrix element *μ*_*i*_. Therefore, to leading order:

32 $$\ln ({\mu }_{i})\propto -| {\delta }_{i}| {\tau }_{i},$$

where *τ*_*i*_ is the temporal width of the trapped state (see Fig. 2h as well as inset to Fig. 5a).

Solving Eq. (30) for *a*_*i*_(*z*) using the Laplace transform (see Sec. I of Supplementary for details), we obtain Eq. (2), with

33 $$\Gamma (z)={{{\mathcal{L}}}}^{-1}\left[\Gamma (s)\right],\Gamma (s)=i\int\,\frac{| {\mu }_{i\omega }{| }^{2}}{is+{\beta }_{\omega }}d\omega,$$

where ${{\mathcal{L}}}$ and ${{{\mathcal{L}}}}^{-1}$ are the Laplace and inverse Laplace transforms. The nonlocal response function is given by:

34 $$\chi (z,{z}^{{\prime} })=\frac{1}{\sqrt{2\pi |{\beta }_{1}|}}\int\,{\mu }_{i\omega }{e}^{i{\beta }_{\omega }z-i\omega {\beta }_{1}{z}^{{\prime} }}{{\rm{d}}}\omega,$$

35 $$p({z}^{{\prime} })=-i\sqrt{\frac{|{\beta }_{1}|}{2\pi }}\int\,{e}^{i\omega {\beta }_{1}{z}^{{\prime} }}{a}_{\omega }(0){{\rm{d}}}\omega .$$

Here and below, we use the denotation *β*_1_ = *β*_1_(*ω*_*i*_).

A local form of these expressions (with *χ* being a constant) is obtained by neglecting all dispersion terms beyond the first order. In this case:

36 $$p(z)=-i\sqrt{{\beta }_{1}}{\left.A(t)\right\vert }_{t={-\beta }_{1}z},\chi=\sqrt{2\pi /|{\beta }_{1}|}{\mu }_{i},$$

where $A(t)={{{\mathcal{F}}}}^{-1}[{a}_{\omega }(0)]$ is the pump wavepacket at *z* = 0. The local version of *Γ*(*z*) (i.e., $\Gamma (z)={{\rm{const}}}$) is obtained by neglecting the *s*-dependence in Eq. (33). This gives, using the Sokhotski-Plemelj formula (see Sec. I of Supplementary for more details):

37 $$\Gamma=\frac{\pi | {\mu }_{i}{| }^{2}}{|{\beta }_{1}|}.$$

The scalars *Γ* and *χ* from Eqs. (37, 36) define thus the local version of the evolution equation (Eq. (2) in the main text), which now reads:

38 $${\partial }_{z}a(z)=-i\kappa a(z)-\Gamma a(z)+\chi p(z).$$

This equation, together with Eqs. (37, 36), describes the standard reciprocal dynamics with *N* = 1.

However, we observe that this local approximation does not hold for the parameter regime considered in the article. Namely, calculations of higher-order terms in *s* (see Sec. I of Supplementary) show that they significantly exceed the leading term in amplitude, clearly indicating a breakdown of the perturbative expansion. Consequently, the decay values obtained in our numerical simulations are several orders of magnitude larger than those predicted by Eq. (37). The reason is the exponentially sensitive dependence of *μ*_*iω*_ on frequency (cf. Eq. (32); See Sec. I have Supplementary for more details).

Alternatively^69^, one can apply the Wentzel-Kramers-Brillouin (WKB) approximation^70^, wherein this decay process can be understood as a tunneling process in the frequency domain, with *δ*_*n*_ determining the width of the tunneling barrier. In^69^, the WKB approximation was very recently used to analyze the case of the same dispersion but somewhat different parameters, giving an estimate which is very close to numerics.

### Linear losses for the trapped states

Even if we have zero leakage through the walls, the losses for the trapped state are not exactly zero. Dissipation of the trapped state takes place due to losses, which include material losses, but also scattering on sub-wavelength-scale roughness of the waveguide material inside the waveguide or on its walls. These scattering-induced losses add to the material losses, and these joint losses are typically measured in experiments as overall “propagation losses”. Achieving very low propagation losses requires advanced waveguide fabrication techniques. In the case of optical fibers, both solid-core and hollow-core fibers are very commonly produced by a technique known as drawing: a relatively short, thick preform is pulled to form a thin, long waveguide with lengths up to many kilometers^71^. As it was already mentioned in the main text, in silica-based waveguides these losses can be as small as 0.1 dB/km ^38–40^, whereas for novel hollow-core gas-filled waveguides they can be even smaller, 0.01 dB/km and less^41^. Even if we use the largest of these two values for our estimation, this gives us the propagation distances of a few km, meaning a lifetime of tens of microseconds and a Q-factor of order of 10^10^. To these losses we must also add dissipation due to perturbations, happening on the larger, multi-wavelength scale, which are considered in the section below.

### Dissipation due to waveguide imperfections

Here, we discuss perturbations on the larger, multi-wavelength scale, such as modifications of the waveguide geometry and refractive index, appearing due to the production-induced imperfections. Such perturbations lead to relatively slow, weak changes of the waveguide geometry and refractive index, and therefore of the soliton shape during propagation. Such a modification can be described as a *z*-dependent stochastic perturbation. *z*-dependence leads to the coupling of different wavevectors in the system, in particular, coupling of the bound states (with the wavenumber *κ*_*n*_) to the continuum states (with *κ*_*f*_ > 0, cf. Fig. 3a of the main text). In more details, for such a perturbation we can derive an analog of Fermi Golden Rule, giving us the losses (derivation details are given in Supplementary, Sec. II):

39 $${\Gamma }_{n}=2\pi \int\,\,d{\kappa }_{f}\rho ({\kappa }_{f})| {A}_{fi}({\kappa }_{f}){| }^{2}S\left({\kappa }_{f}-{\kappa }_{n}\right),$$

where *S*(*κ*) is the power spectral density of the perturbation, ∣*A*_*fi*_(*κ*_*f*_)∣^2^ is the amplitude of the perturbation, *ρ*(*κ*_*f*_) is the density of the continuum states.

If the waveguides are produced using the drawing process mentioned above in the previous section, these inhomogeneities happen on the scale of 1−100 meters^72,73^, translated into wavevectors uncertainties on the scale of *σ* ~ 10^−6^ − 10^−8^ μm^−1^. This is much less than the typical values of *κ*_*n*_ for the bound states, which are of the order of *κ*_*n*_ ~ 10^−1^ − 10^−2^ μm^−1^ (cf. Fig. 3a). This means that such perturbations leave the bound states essentially decoupled from the continuum, making dissipation, induced by such perturbations, negligible.

Indeed, in more details, let us make very reasonable assumption for the perturbation to be Gaussian, that is,

40 $$S(\kappa )={S}_{0}\exp \left[-\frac{{\kappa }^{2}}{2{\sigma }^{2}}\right]$$

with some amplitude *S*_0_ and with *σ*. Taking into account the numbers above, the exponent in this Gaussian is of the order of 10^−100^, meaning the coupling of the bound and continuum states is essentially negligible by any reasonable perturbation amplitudes, and thus dissipation due to this mechanism is also negligible.

Note that this consideration is not universally valid. It may fail for the states which are very close to the continuum (halo states), but also for other waveguide types, such as slot waveguides. For the latter, the typical scale for uncertainties is much smaller (millimeters and centimeters), leading to significantly higher leakage (on the other hand, such waveguides have typically also much lower length, also in the centimeter range, as well as much higher losses). Nevertheless, the consideration above shows that there are waveguide types for which the trapping can be very effective.

### Potential shapes deviating from the sech-shape

Now let us consider how the trapped states and their eigenvalues, analytically known for the sech-shaped potential (see section “Trapped States” above), are modified when the potential is “deformed” under the action of additional effects such as self-steepening or higher-order dispersion.

Self-steepening technically manifests itself in the presence of frequency dependence of the nonlinear coefficient. In general, the effect of self-steepening is twofold. First, the modification of the ratio *γ*(*ω*_*s*_)/*γ*(*ω*_*t*_) in Eqs. (10, 11) leads to a modification in the number and wavevectors of the trapped states. But beyond this, the remaining changes to the wavefunctions, caused by the modification of the potential well due to the intrapulse frequency dependence *γ*(*ω*), are rather weak and lead to a deformation of the trapped state, which can be calculated using standard perturbation theory^74^:

41 $${k}_{n}\to {k}_{n}+{\delta }_{nn}+{\sum}_{i\ne n}\frac{| {\delta }_{in}{| }^{2}}{{k}_{n}-{k}_{i}},$$

42 $${\psi }_{n}(t)\to {\psi }_{n}+{\sum}_{i\ne n}\frac{{\delta }_{ni}}{{k}_{n}-{k}_{i}}{\psi }_{i}(t),$$

where we assume a perturbation of the potential *V*(*t*) → *V*(*t*) + *δV*(*t*), with matrix elements

43 $${\delta }_{in}=\int\,{\psi }_{i}(t)\delta V(t){\psi }_{n}^{*}(t)dt.$$

A less standard case is a perturbation of the dispersion: $\hat{\beta }\to \hat{\beta }+\hat{\delta }$, with $\hat{\delta }$ now being the dispersion perturbation. As long as this does not introduce new resonances (i.e., frequencies for which *β*(*ω*) = -*κ*_*n*_), the same perturbation theory applies, using the same formulas as above, with

44 $${\delta }_{in}=\int\,{\psi }_{i}(t)\hat{\delta }{\psi }_{n}^{*}(t)dt.$$

In contrast, if new resonances are introduced, the corresponding eigenfunctions are no longer strictly bound, as they become coupled to radiation. This case is discussed above in the section “Equations for the Amplitudes”.

### Time-bandwidth limit

Here, as well as throughout the article, we assume a plane wave of the form $\exp (-i\omega t+ikz)$. This convention results in opposite signs for the phase factors in the time- and space-propagated equations. A general stationary single-mode cavity with a single output channel, pump *p*, and losses is governed by Eq. (3) or its vectorial extensions^31,33,75,76^. The frequency-domain solution of Eq. (3) is

45 $$a(\omega )=\frac{-i\chi p(\omega )}{(\kappa-\omega)-i\Gamma }.$$

This corresponds to a Lorentzian spectral line centered at the resonance frequency *κ*, with spectral density

46 $$| a(\omega ){| }^{2}\propto \frac{1}{{(\kappa-\omega)}^{2}+{\Gamma }^{2}},$$

indicating a FWHM width of *Δω* = 2*Γ*. Since the field decays as ∣*a*∣ ∝ *e*^−*Γt*^, the decay time is *Δt* = 1/*Γ*, leading to the time-bandwidth product

47 $$M\equiv \Delta \omega \Delta t=2.$$

We note that, while the exact numerical value on the left-hand side may depend on the specific definitions of linewidth (FWHM vs. 1/*e*, etc.) and decay (amplitude vs. intensity) ^31,33^, the value of *M* remains, by any common definition, on the order of one.

In contrast, in our case of a time-trap, the dynamics follow Eq. (2) rather than Eq. (3), describing spatial rather than temporal evolution, and introducing nonlocality. For $\chi (z,{z}^{{\prime} })=\chi (z-{z}^{{\prime} })$, this equation is solved in *k*-space as:

48 $$a(k)=\frac{i\chi (k)p(k)}{(\kappa+k)+i\Gamma (k)},$$

where *k* is the spatial wavevector and *χ*(*k*), *Γ*(*k*), *p*(*k*) are the Fourier transforms of their respective spatial-domain quantities. While Eq. (48) is again a Lorentzian, this is the Lorentzian in *k*-space rather than in frequency. Therefore, it does not directly define the spectral bandwidth. Instead, the frequency-domain bandwidth is governed by the trapped state spectrum *Ψ*_*i*_(*ω*), and thus by the soliton duration *Δω* ~ 1/*τ*_*s*_ (cf. Eqs. (13–17)). As a result, in this spatially-propagated setting, *Γ* and *Δω* are completely decoupled and can differ by orders of magnitude. They can also be varied independently.

### Limit on energy loading from dispersive wave to the trapped state

In most standard cases, cavities governed by Eq. (3) obey energy conservation and time-reversal symmetry, leading to reciprocity: the coupling constants for input (*χ*) and output (*Γ*) are related by *N* = 1 (see main text and Sec. III of Supplementary, in particular Supplementary Figs. 1 and 2). We estimate the upper bound on energy loading efficiency *ϵ* = *W*_in_/*W*_pump_, where *W*_in_ is the energy stored in the cavity after pump passage, and *W*_pump_ is the initial pulse energy.

Neglecting the loss term *Γa*, integration of Eq. (3) gives

49 $$a=\int_{0}^{+\infty }\chi \,p(t)dt.$$

Taken into account normalizations mentioned above,*W*_in_ = ∣*a*∣^2^, *W*_pump_ = ∫∣*p*(*t*)∣^2^*dt*, and we obtain

50 $$\epsilon={\chi }^{2}F\tau,\quad F=\frac{({\int}f(t)dt/\tau )^{2}}{\int\,{f}^{2}(t)dt/\tau },$$

where *τ* is the pulse duration and f(t)=p(t)/max[p(t)] describes the pulse shape. Using the definition of *N* from the main text, this yields Eq (4).

Next, consider the same limit for the local time-cavity model Eq. (38). Rewriting with *t* = *z*/*v*_*s*_, we obtain

51 $${\partial }_{t}a(t)=-i\frac{\kappa }{{v}_{s}}a(t)-\frac{\Gamma }{{v}_{s}}a(t)+\frac{\chi }{\sqrt{{v}_{s}}}p(t),$$

where *p*(*z*) is rescaled to $p(t)/\sqrt{{v}_{s}}$ (see Sec. I of Supplementary). Reciprocity is preserved under this transformation, so *N* = 1 remains valid.

Integrating Eq. (51) yields *ϵ* in the same form as Eq. (4), but with an increased effective interaction time: instead of the pump duration *τ*, we now have *τ*/(*v*_*g*_*β*_1_). We can thus absorb this additional factor 1/(*v*_*g*_*β*_1_) into *N*, replacing *N* = 1 with *N* = *N*_geom_ = 1/(*v*_*g*_*β*_1_), which we refer to as geometric effective nonreciprocity.

Finally, in our full model, both *χ* and *Γ* are nonlocal operators rather than constants. Despite this, we can numerically determine the energy transferred from the dispersive wave to the trapped state. Comparing this to the analytic upper limit in Eq. (4), we observe an additional enhancement to *ϵ*, which we define as *N*_nonlocal_ and incorporate into *N* accordingly.

### Transmission delays

In traditional spatial potentials, delays are measured in time. In contrast, for a time-dependent potential, delays are naturally measured in propagation distance *z*. To quantify the *z*-duration for loading and re-emission of radiation into and from the solitary-wave trap, we adapt the concept of quantum mechanical dwell time^77,78^.

Let *P*_*T*_(*z*) be the fraction of the dispersive wave’s energy located within the domain (−*T*/2, *T*/2) at propagation distance *z*. Then the dwell distance is

52 $${z}_{{{\rm{D}}}}=\int\,{P}_{T}(z)\,dz.$$

The domain is centered on the trapping soliton and has extent *T* = 4*τ*_*s*_. The corresponding free-propagation dwell distance (in the absence of the soliton) is approximately *z*_free_ ≈ *τ*_*s*_/*β*_1_, where *β*_1_ is the inverse group velocity of the dispersive wave in the soliton’s co-moving frame.

## Supplementary information


Supplementary Information
Transparent Peer Review file


## Data Availability

All data supporting the findings of this study are available at Zenodo (https://doi.org/10.5281/zenodo.21493595).

## Code availability

Simulations in the article were made using the software package FXMAS^79^, available at Code Ocean (https://codeocean.com/capsule/8221780) and Github (https://github.com/omelchert/py-fmas). The code reproducing some of the key figures of the article is available at Code Ocean (https://doi.org/10.24433/CO.0788432.v1). The rest is available from the corresponding author upon request.
