# Supplementary material for: Photon trapping in a time cavity flying at the speed of light
Source: Nat Commun. 2026 Aug 29;17:9235. doi: 10.1038/s41467-026-77059-1 (PMC13526024; doi:10.1038/s41467-026-77059-1)
Supplement: Supplementary file 1 [file 41467_2026_77059_MOESM1_ESM.pdf]

Photon trapping in a time cavity flying at the speed of light: Supplementary Material

Ihar Babushkin,[1,2] Oliver Melchert,[1,2] Uwe Morgner,[1,2] and Ayhan Demircan[1,2,3]
[1] *Cluster of Excellence PhoenixD, Welfengarten 1, 30167, Hannover, Germany*
[2] *Institute of Quantum Optics, Leibniz Universität Hannover, Welfengarten 1, 30167, Hannover, Germany*
[3] *Hannover Centre for Optical Technologies, Nienburger Str. 17, 30167, Hannover, Germany*
(Dated: August 25, 2026)

I. AMPLITUDE EQUATIONS: EXTENDED DISCUSSION

A. Definition of the operators

Here, following the analogy to the standard quantum mechanics we call our acting operators "Hamiltonians". They are more general than typical Hamiltonians in the standard quantum mechanics (see Methods for more details), yet our dynamics is unitary, so the very standard techniques of quantum mechanics are fully applicable. To derive the coupled equations for the amplitudes, we need to define a pair of Hamiltonians $\hat{H}_0$ and $\hat{W}$ such that their sum give us our full Hamiltonian $\hat{H}$, whereas $\hat{H}_0$ catches the bound states and $\hat{W}$ the free states. It might appear to be natural to consider $\hat{H}_0 = (\beta_2/2)\partial_{tt} + V(t)$, $\hat{W} = -\hat{\beta} - \hat{H}_0$. However, in this situation the eigenvalues (wavevectors) of the free (non-trapped) waves will be modified significantly; this, in particular, prevents from the phase matching between the trapped states and the free states, which is what we actually want to describe here. To circumvent this difficulty we introduce a function $U(t)$ which has the following properties: $U(t) = 1$ in the vicinity of $t = 0$, much larger than the duration of the trap $V(t)$. We furthermore request that $U(t) = 0$ for large enough $|t|$. We will call the regions where $U = 1$ and $U = 0$ the support and the complement of the support, correspondingly. Besides, $U(t)$ must change slowly enough comparing with all frequencies ω we are interested in, so that the incoming waves $\sim e^{-i\omega t}$, instead of being reflected, adiabatically change their wavevector as the wave enter the region where $U(t) \neq 0$. The particular shape of $U(t)$ is not important. Such functions $U(t)$, even fully analytical, exist. As a result, we obtain the decomposition mentioned in Methods.

If the support of U is large enough, the trapped eigenfunctions and the corresponding eigenvalues are close to the eigenfunctions $\Psi_i(t)$ and eigenvalues κ_i of $\hat{H}_0$ considered analytically in Methods, since, near the location of the potential $V(t)$, $H \approx H_0$. On the other hand, far away from $V(t)$ we have $H \approx \hat{-\beta}$ and therefore is very different from H_0, but there the eigenfunctions are negligible (since they decay exponentially with t), therefore the form of the Hamiltonian away from $V(t)$ does not influence the shape of the eigenfunctions. The non-trapped states with wavefunctions $\Psi_\omega(t)$ have at $|t| \to \infty$ the asymptotic shapes $\Psi_\omega(t) \sim e^{-i\omega t}$ and their eigenvalues must be equal to $-\beta(\omega)$. The matrix elements $\mu_{i\omega} = \int \Psi_i^*(t)\hat{W}\Psi_\omega(t)dt$ and $\mu_{\omega i} = \int \Psi_\omega^*(t)\hat{W}\Psi_i(t)dt$ are therefore good approximated by Eq. 21 of Methods. To calculate these matrix element, the eigenfunctions of $\hat{H}_0$ (see the discussion in "Trapped States" in Methods) can be used as a suitable approximation. The unitary nature of the problem imposes $\mu_{i\omega} = \mu_{\omega i}^*$.

B. Equations for the modes: details of the derivation

As stated in the Methods, we consider the expansion in the form

$$\Psi(t, z) = \sum_i a_i(z)\Psi_i(t) + \sum_\omega a_\omega(z)\Psi_\omega(t). \tag{1}$$

To avoid nonphysical divergences, we formally embed our system into a large box (in time) of size $\mathcal{T}$, which renders the spectrum of non-trapped states discrete. Similar to other analogous problems, such as spontaneous emission, $\mathcal{T}$ will cancel out in the final expressions.

Because of the unitary nature of the problem, the eigenstates of H_0 are orthogonal. We normalize the trapped states of H_0 to the trapped energy and the non-trapped states to intensity, resulting in the following orthonormality relations:

$$\int \Psi_i^*\Psi_j \, dt = \delta_{ij}, \int \Psi_\omega^*\Psi_{\omega'} \, dt = (\mathcal{T}/2\pi)\delta_{\omega\omega'},$$
$$\int \Psi_i^*\Psi_\omega \, dt = 0.$$

Projecting onto each of the eigenstates, we obtain the equations for the partial amplitudes:

$$i\partial_z a_i = \kappa_i a_i + \sum_\omega \mu_{i\omega} a_\omega, \quad i\partial_z a_\omega = -\beta_\omega a_\omega + \frac{2\pi}{\mathcal{T}}\mu_{\omega i} a_i, \tag{2}$$

with $\mu_{i\omega}$ defined above.

As the next step, we restrict our consideration, for simplicity, to a single trapped state i, replace sums with integrals as $\sum_\omega(\cdot) \to (\mathcal{T}/2\pi)\int(\cdot)\, d\omega$, and redefine the amplitudes as $(\mathcal{T}/2\pi)\, a_\omega \to a_\omega$, thus finally obtaining Eq. 20 in the Methods section of the main article:

$$i\partial_z a_i = \kappa_i a_i + \int \mu_{i\omega} a_\omega \, d\omega, \quad i\partial_z a_\omega = -\beta_\omega a_\omega + \mu_{\omega i} a_i. \tag{3}$$

C. Cavity equation (Eq. 2 of the main article): details of the derivation

We can integrate equations Eq. (3) using Laplace transform $\mathcal{L}$, which for every function $f(z)$ represents its image $f(s)$. It gives us (assuming for simplicity $\kappa_i = 0$):

$$isa = \int \mu_{i\omega} a_\omega \mathrm{d}\omega, \tag{4}$$

$$isa_\omega = -\beta_\omega a_\omega + \mu_{\omega i} a + i a_\omega(0), \tag{5}$$

where now denote $a = a(s)$, $a_\omega = a_\omega(s)$, $a_\omega(0) = a_\omega(z)|_{z=0}$, and we assumed in addition that $a(z)|_{z=0} = 0$, that is we have the empty trap before the pump pulse comes. The second equation is resolved as:

$$a_\omega = \frac{\mu_{\omega i} a + i a_\omega(0)}{is + \beta_\omega}. \tag{6}$$

Substituting it into Eq. (4), we get:

$$sa = \frac{1}{i} \int \frac{|\mu_{i\omega}|^2 a}{is + \beta_\omega} \mathrm{d}\omega + \int \frac{\mu_{i\omega} a_\omega(0)}{is + \beta_\omega} \mathrm{d}\omega. \tag{7}$$

We can now make the inverse Laplace transform $\mathcal{L}^{-1}$ of this expression, taking into account the convolution theorem (multiplication of the Laplace images results in the convolution in the real space). The first term on the right hand side gives us then:

$$-\int\limits_0^z \Gamma(z - z') a(z') dz', \tag{8}$$

where $\Gamma(z)$ is defined as:

$$\Gamma(z) = \mathcal{L}^{-1}\left[\Gamma(s)\right], \Gamma(s) = i \int \frac{|\mu_{i\omega}|^2}{is + \beta_\omega} d\omega. \tag{9}$$

To calculate the second, inhomogeneous, term on the right hand side in Eq. (7), we follow the known properties of the Laplace transform: for an arbitrary Laplace image $F(s)$ we have

$$\mathcal{L}^{-1}\left[\frac{F(s)}{s + i\alpha}\right](z) = e^{-i\alpha z} \mathcal{L}^{-1}\left[\frac{F(s - i\alpha)}{s}\right](z)$$
$$= e^{-i\alpha z} \int\limits_0^z e^{i\alpha z'} \mathcal{L}^{-1}\left[F(s)\right](z') \mathrm{d}z'. \tag{10}$$

We apply these transformations for each frequency ω separately in the second term on the right-hand side of Eq. (7), taking $\alpha = -\beta_\omega$ and $F(s) = -\mu_{i\omega} a_\omega(0)$. The latter, in particular, means that $F(s)$ does not depend on s, i.e., $F(s) = \mathrm{const}(z)$, and thus $\mathcal{L}^{-1}\left[F(s)\right](z') = \delta(z')$. This allows us to obtain the inhomogeneous term in Eq. (7) as:

$$-i \int a_\omega(0) \mu_{i\omega} e^{i\beta_\omega z} \mathrm{d}\omega. \tag{11}$$

We now introduce the pump $p(\omega)$ and pump-to-cavity coupling $\chi(z, \omega)$, as well their Fourier transforms $\chi(z, t)$, $p(t)$ so that their convolution reproduce this term Eq. (11):

$$-i \int a_\omega(0) \mu_{i\omega} e^{i\beta_\omega z} \mathrm{d}\omega = \int \chi(z, \omega) p(\omega) \mathrm{d}\omega$$
$$= \int \chi(z, t) p(t) \mathrm{d}t. \tag{12}$$

For this, we define χ and p as

$$\chi(z, \omega) = \frac{1}{\sqrt{|\beta_1|}} \mu_{i\omega} e^{i\beta_\omega z}, \tag{13}$$

$$p(\omega) = -i\sqrt{|\beta_1|} a_\omega(0), \tag{14}$$

where, as given in the Methods in the main text, we denote $\beta_1(\omega_i)$ as β_1. Here, $p(\omega) \propto a_\omega(0)$, that is, the initial condition for the dispersive wave at $z = 0$. Note, that here we also introduced the factor $\sqrt{|\beta_1|}$ into the definition of $p(\omega)$. This factor takes into account the change of the definition of energy density of the pump in the moving frame of reference and will be discussed in the next section. The reciprocal factor $1/\sqrt{|\beta_1|}$ appears then in the definition of χ to ensure that Eq. (12) holds. Making the inverse Fourier transform $\omega \to t$ in Eqs. (13)-(14), we obtain:

$$\chi(z, t) = \frac{1}{\sqrt{2\pi|\beta_1|}} \int \mu_{i\omega} e^{i\beta_\omega z + i\omega t} \mathrm{d}\omega, \tag{15}$$

$$p(t) = -i\sqrt{\frac{|\beta_1|}{2\pi}} \int e^{-i\omega t} a_\omega(0) \mathrm{d}\omega. \tag{16}$$

In the rest of this subsection, we will transform this term into the form $\int \chi(z, z') p(z') \mathrm{d}z'$ (with both arguments being propagation distances), which it has in Eq. 2 of the main article. We obtain the term with $p(z')$ in Eq. 2 of the main article by replacing t by $\beta_1 z'$, in Eqs. (15)-(16), that is, introducing $\chi(z, z')$ and $p(z')$ in such a way that

$$\int \chi(z, t) p(t) \mathrm{d}t = \int \chi(z, z') p(z') \mathrm{d}z', \tag{17}$$

with:

$$\chi(z, z') = \chi(z, t)|_{t=-\beta_1 z'}, \tag{18}$$

$$p(z') = p(t)|_{t=-\beta_1 z'}, \tag{19}$$

that is,

$$\chi(z, z') = \frac{1}{\sqrt{2\pi|\beta_1|}} \int \mu_{i\omega} e^{i\beta_\omega z - i\omega\beta_1 z'} \mathrm{d}\omega, \tag{20}$$

$$p(z') = -i\sqrt{\frac{|\beta_1|}{2\pi}} \int e^{i\omega\beta_1 z'} a_\omega(0) \mathrm{d}\omega, \tag{21}$$

which is are expressions, shown in the Methods: These definitions are specially straightforward to understand,

if during the collision time (of the dispersive wave with the soliton) the dispersive broadening is negligible (the typical situation presented in Figs. 4,5 of the main article). In this case, the light propagates mostly along the rays $t = \beta_1 z$, so that the replacement above is fully satisfied. However, we note that the definitions above are fully allowed even in more general cases where dispersive broadening plays a role, although they lose their straightforward meaning in such cases.

Now, let us return to Eq. (7). Collecting all the pieces together, we obtain:

$$\partial_z a = -\int \Gamma(z - z')a(z')dz' + \int \chi(z, z')p(z')dz', \quad (22)$$

which is basically Eq. 2 of the main article (in the latter we additionally taken into account the possible nonzero eigenvalue κ).

D. The local variant of the cavity equation, and breaking of the perturbative approximation

Let is study the conditions, when the nonlocal expression, derived in the previous subsection, can be converted into a local expression, that is, when we have in Eq. (22):

$$\Gamma(z) \propto \delta(z),\ \chi(z, z') \propto \delta(z - z'), \quad (23)$$

The former of these two equations assumes that

$$\Gamma(s) \propto \mathrm{const}(s) \quad (24)$$

in Eq. (9). Thus, as it was mentioned in the Methods, we can try to obtain the analytical expression for $\Gamma(s)$ by neglecting s in Eq. (9). The corresponding integral can be calculated using the Sokhotski–Plemelj formula. Assuming for simplicity $\beta_\omega \approx \beta_1(\omega - \omega_i) = \beta_1\Omega$, and using:

$$\lim_{\epsilon\to+0} \frac{1}{\beta_1\Omega \pm i\epsilon} = \mp i\pi\delta(\beta_1\Omega) + \mathcal{P}\left(\frac{1}{\beta_1\Omega}\right), \quad (25)$$

where $\mathcal{P}$ denotes the Cauchy principal value, and $\Omega = \omega - \omega_i$, since we have resonance $\beta_\omega = 0$ at $\omega = \omega_i$. We can furthermore assume that $|\mu_{i\omega}|$ is symmetric in respect to this resonance (has a maximum at $\omega = \omega_i$), so that the Cauchy principal value is zero. Taking into account that $\delta(\beta_1\Omega) = \delta(\Omega)/|\beta_1|$, we obtain the expression given in Methods:

$$\Gamma = \pi|\mu_i|^2/|\beta_1|. \quad (26)$$

Now, let us consider the pump term in Eq. (22), which we here consider in the form $\int \chi(z, \omega)p(\omega)\mathrm{d}\omega$ as given by Eq. (12). Assuming that $a_\omega(0)$ is a wavepacket, centered at ω_i in frequency domain and at time τ in time domain for $z = 0$, that is, having the form $= A(\Omega)e^{-i\omega\tau}$ for some slow envelope $A(\Omega)$, and neglecting the frequency dependence of $\mu_{i\omega}$, that is $\mu_{i\omega} \approx \mathrm{const}(\omega) \approx \mu_i$, one can rewrite the second term in right side in Eq. (22) as

$$-i\mu_i \int A(\Omega)e^{i\Omega\beta_1 z}d\Omega = -i\sqrt{2\pi}\mu_i A(t)\,|_{t=-\beta_1 z}\ , \quad (27)$$

where $\Omega = \omega - \omega_i$, $A(t)$ is the inverse Fourier transform of $A(\Omega)$. By denoting

$$\chi = \sqrt{\frac{2\pi}{|\beta_1|}}\mu_i,\ p(z) = -i\sqrt{|\beta_1|}A(t)\,|_{t=-\beta_1 z}\ , \quad (28)$$

we obtain a local variant of the nonlocal equation Eq. (22)

$$\frac{\mathrm{d}a(z)}{\mathrm{d}z} = -\Gamma a(z) + \chi p(z), \quad (29)$$

with Γ and χ being the numbers given by Eq. (26), Eq. (28). As one can see, these local consideration leads to $N = 1$. This, we confirmed, that in the case, when s is negligible, that is, the dynamics is slow enough, and when the dispersion of higher order is negligible for the pump, we obtain fully reciprocal local expression Eq. (29).

Nevertheless, Γ given by Eq. (26) for the parameters of Fig. 4 of the main article is around 10^{-8} μm^{-1}, which is several orders of magnitude smaller than the value $\sim 10^{-4}$ μm^{-1} predicted by numerical simulations. To understand the origin of this discrepancy, we calculate the influence of $\Gamma(s)$ introduced by the frequency dependence of $|\mu_i|^2$.

To this end, we assume that ω' is the (complex) root of the denominator in Eq. (9), i.e., the solution of $is+\beta_{\omega'} = 0$. We define $\delta\omega = \omega' - \omega_i$ as the shift with respect to the unperturbed root ω_i for $s \to 0$, obtaining:

$$\delta\omega \approx \frac{2is}{\beta_1\beta_2}. \quad (30)$$

This approximation is obtained by including dispersion up to second order, expanding the resulting root in powers of s, and retaining the solution that vanishes as $s \to 0$. The shift $\delta\omega$ effectively modifies the frequency at which μ_i is evaluated. As a result, μ_i is corrected as

$$|\mu_i|^2 \equiv |\mu_{i\omega_i}|^2 \to |\mu_{i\omega_i+\delta\omega}|^2 \approx |\mu_{i\omega_i}|^2 + \delta\omega\,\partial_{\omega_i}|\mu_{i\omega_i}|^2. \quad (31)$$

The corresponding modification $\delta\Gamma(s)$ of $\Gamma(s)$, arising from the perturbation term in this expansion, is computed analogously to the original term (still assuming $\omega = \omega_i$ in the denominator of Eq. (9)), yielding:

$$\delta\Gamma(s) \approx -\frac{\pi s}{\beta_1^2\beta_2}\frac{\partial|\mu_i|^2}{\partial\omega_i} + \ldots \quad (32)$$

This correction term is approximately four orders of magnitude larger than the value predicted by Eq. (26), even when assuming the "minimal possible" value of $|s|$ given by the zeroth-order estimate of Γ in Eq. (26). This clearly indicates that, under our parameter regime, the

system is far beyond the perturbative regime. The analysis above shows that the main effect leading to this discrepancy is the sensitivity of μ_i to frequency perturbations. Therefore, we conclude that the local form of the equation with Γ given by Eq. (26) cannot be used as a first-order approximation.

II. FERMI GOLDEN RULE FOR STOCHASTIC PERTURBATIONS ALONG z

Here we develop perturbation theory for considering perturbation in the waveguide form, as the trap (soliton) propagates in the waveguide. This perturbation theory will be very similar to the one developed for the traditional Schrödinger equation (with replacing $\hbar \to 1$, $t \to z$), therefore we will use traditional denotations (somewhat different from the denotations in the rest of the text). In the bra-ket notation, our basic linear equation, describing trapping and the evolution along z, can be written as

$$i\partial_z \left|\psi(z)\right\rangle = \hat{H} \left|\psi(z)\right\rangle, \tag{33}$$

governed by the Hamiltonian

$$H(z) = H_0 + V(z), \tag{34}$$

where in this section we denote H_0 the unperturbed Hamiltonian. Its eigenstates we denote in this section as $|n\rangle$ and the corresponding propagation constants κ_n, and $V(z)$ is a weak z-dependent perturbation. Suppose the system is initially in the bound sate state $|i\rangle$. We want to determine the transition probability to some final state $|f\rangle$, particularly when $|f\rangle$ belongs to a continuum. We develop the corresponding analog of Fermi Golden rule and estimate the escape rate for the trapped state, governed by the perturbation.

We furthermore suppose for the sake of simplicity

$$V(z) = A\,\xi(z), \tag{35}$$

A is a fixed operator, and $\xi(z)$ is a stationary classical random process with zero mean,

$$\langle \xi(z) \rangle = 0, \tag{36}$$

and correlation function

$$C(\delta z) = \langle \xi(z+\delta z)\,\xi(z) \rangle. \tag{37}$$

In the interaction picture,

$$V_I(z) = e^{iH_0 z} V(z) e^{-iH_0 z} = A_I(z)\,\xi(z), \tag{38}$$

with

$$A_I(z) = e^{iH_0 z} A e^{-iH_0 z}. \tag{39}$$

To first order in the perturbation,

$$|\psi_I(z)\rangle = |i\rangle - i \int_0^z dz_1\, V_I(z_1)\,|i\rangle + O(V^2). \tag{40}$$

The probability amplitude to be in $|f\rangle$ at position z is

$$c_f(z) = -i \int_0^z dz_1\, \langle f|\, A_I(z_1)\, |i\rangle\; \xi(z_1). \tag{41}$$

Since

$$\langle f|\, A_I(z)\, |i\rangle = A_{fi}\, e^{i\kappa_{fi} z}, \qquad \kappa_{fi} = \kappa_f - \kappa_i, \tag{42}$$

we obtain

$$c_f(z) = -iA_{fi} \int_0^z dz_1\, \xi(z_1)\, e^{i\kappa_{fi} z_1}. \tag{43}$$

The transition probability is

$$P_{i\to f}(z) = \langle |c_f(z)|^2 \rangle. \tag{44}$$

Using the expression above,

$$\begin{aligned} P_{i\to f}(z) &= |A_{fi}|^2 \int_0^z dz_1 \int_0^z dz_2\, \langle \xi(z_1)\xi(z_2) \rangle\, e^{i\kappa_{fi}(z_1 - z_2)} \\ &= |A_{fi}|^2 \int_0^z dz_1 \int_0^z dz_2\, C(z_1 - z_2)\, e^{i\kappa_{fi}(z_1 - z_2)}, \end{aligned} \tag{45}$$

where stationarity of the noise was used: $\langle \xi(z_1)\xi(z_2) \rangle = C(z_1 - z_2)$.

For long propagation distances z the integration domain may be approximated by a rectangle, yielding:

$$P_{i\to f}(z) \simeq |A_{fi}|^2\, z \int_{-\infty}^{+\infty} dz'\, C(z')\, e^{i\kappa_{fi} z'}. \tag{46}$$

We furthermore define the noise power spectral density as:

$$S_\xi(\kappa) = \frac{1}{2\pi} \int_{-\infty}^{+\infty} dz'\, C(z')\, e^{i\kappa z'}. \tag{47}$$

Thus

$$\int_{-\infty}^{+\infty} dz'\, C(z')\, e^{i\kappa_{fi} z'} = 2\pi\, S_\xi(\kappa_{fi}). \tag{48}$$

Hence the transition probability becomes

$$P_{i\to f}(z) \simeq 2\pi\, |A_{fi}|^2\, S_\xi(\kappa_{fi})\, t. \tag{49}$$

The transition rate for a single final state is therefore

$$\Gamma_{i\to f} = \frac{P_{i\to f}(z)}{z} = 2\pi\, |A_{fi}|^2\, S_\xi(\kappa_{fi}). \tag{50}$$

If the final states form a continuum with density of states $\rho(\kappa_f)$, the total transition rate out of the initial state $|i\rangle$ is

$$\Gamma_i = \int d\kappa_f\, \rho(\kappa_f)\, \Gamma_{i\to f}(\kappa_f), \tag{51}$$

which yields

$$\Gamma_i = 2\pi \int d\kappa_f\, \rho(\kappa_f)\, |A_{fi}(\kappa_f)|^2\, S_\xi(\kappa_f - \kappa_i)\,. \tag{52}$$

This is the extension of the Fermi Golden Rule for a z-dependent noisy perturbation for our time-trap. This expression, with small changes in denotations $S_\xi \to S$ is shown in the Methods in the main article.

III. RECIPROCITY

Since we consider the fundamental limits on maximal efficiency, which which the cavity we can be loaded, the fundamental relation between the out- and in-coupling coefficients in the cavity must be considered in more details, in particular the question of reciprocity/nonreciprocity, which we do in this section.

A. Relation between in- and out-coupling coefficients

Typically, the system of equations describing both the pump and the intracavity field obeys the following conditions (see, for instance, [1]):

i) energy conservation,

ii) time reversibility.

Cavities that satisfy these conditions are referred to as reciprocal. The dynamics in traditional reciprocal cavities are typically described by the following equation:

$$\frac{da(t)}{dt} = -\Gamma a(t) + \chi p(t), \tag{53}$$

where a is the amplitude of the intracavity field, p is the external pump, Γ represents the losses, and χ is the coupling coefficient from the pump to the cavity. For simplicity, we assume zero detuning from resonance and neglect all loss channels except the one associated with pump coupling.

In this subsection, we derive—purely for illustrative and completeness purposes—the conditions that reciprocity imposes on the relation between Γ and χ.

For the following, the normalizations of a and p are important. The amplitude a must be normalized to the total energy stored inside the cavity or trap:

$$W_{\text{in}} = |a|^2. \tag{54}$$

The pump field p must be normalized to intensity, such that

$$\int p(t)\, dt = W_{\text{out}}, \tag{55}$$

where W_{out} is the total energy extracted from or delivered to the cavity.

Energy conservation implies that

$$dW_{\text{in}} = -dW_{\text{out}} = -|p|^2\, dt, \tag{56}$$

i.e., the energy lost from a appears in p, and vice versa. This can be rewritten as

$$\frac{dW_{\text{in}}}{dt} = -|p|^2. \tag{57}$$

Now, let's assume that $p(0) = 0$, and $a(0) = a_0$, that is, at $t = 0$, cavity is loaded and no external field is present. The solution of Eq. (53) for this case is simply

$$a(t) = a_0 e^{-\Gamma t}. \tag{58}$$

For $p(t)$ we take into account energy conservation above, which says us that

$$W_{\text{in}}(t) = a_0^2 e^{-2\Gamma t},\ \frac{\mathrm{d}W_{\text{in}}}{\mathrm{d}t} = -2\Gamma a_0^2 e^{-2\Gamma t}, \tag{59}$$

that is, combining with Eq. (57), we have:

$$p(t) = \sqrt{2\Gamma} a_0 e^{-\Gamma t}, \tag{60}$$

Now, we propagate the system forward until some sufficiently long time T, and then perform a time reversal $t \to -t$ and propagate backward from T to 0, which can be represented as the propagation of time-reversed system

$$\frac{\mathrm{d}a(t)}{\mathrm{d}t} = +\Gamma a(t) - \chi p(t), \tag{61}$$

from 0 to T with $p(t)$ given by Eq. (58) with $t \to -t$, and $p(0)$ given by $P(T)$ in Eq. (58), that is: $p(t) = \sqrt{2\Gamma} a_0 e^{\Gamma t - \Gamma T}$. The general solution of the back-propagated equation Eq. (61) at time t is

$$a(t) = -\chi e^{-\Gamma t} \int_0^t p(t') e^{\Gamma t'} dt' - a(0) e^{-\Gamma t} =$$

$$= -\frac{\sqrt{2\Gamma}\chi a_0}{2\Gamma} e^{-\Gamma t - \Gamma T} \left(e^{2\Gamma t} - 1\right) + a_0 e^{-\Gamma t - \Gamma T}, \tag{62}$$

where we have taken into account that $a(0) = a_0 e^{-\Gamma T}$. Substituting $t \to T$ and afterwards $T \to \infty$ and thereby neglecting the terms, exponentially small with T, we will obtain:

$$a(T) = -a_0 \frac{\chi}{\sqrt{2\Gamma}}. \tag{63}$$

Therefore, to have $a(T) = a_0$ (as it should be if our equation is time reversible), we have to have

$$|\chi| = \sqrt{2\Gamma}, \tag{64}$$

which the standard expression for the reciprocity. By introducing N as:

$$N = \frac{\chi^2}{2\Gamma}. \tag{65}$$

We can recast Eq. (64) into the form

$$N = 1, \tag{66}$$

which is used in the main article.

B. Non-reciprocity

Nonreciprocity can appear if the reversibility in time is not anymore present. An example is the situation when magnetic field is present. In this case, the equations driving particles (with coordinate $\mathbf{r}$) are

$$\frac{\mathrm{d}^2\mathbf{r}}{\mathrm{d}t^2} = q(\frac{\mathrm{d}\mathbf{r}}{\mathrm{d}t}) \times \mathbf{B}, \tag{67}$$

where $\mathbf{B}$ is the magnetic field. The left side is invariant under the change of the time direction $t \to -t$. On the other hand, $d\mathbf{r}/dt \to -d\mathbf{r}/dt$. Therefore, in order to maintain the time-reversibility of Eq. (67) as whole, we need to reverse, together with time direction, also the direction of $\mathbf{B}$ ($\mathbf{B} \to -\mathbf{B}$). But what if we reverse the direction of time, keeping $\mathbf{B}$ unchanged? This is what we can do in a real physical system. In the situation when we have certain external, pre-given $\mathbf{B}$, reversing the direction of time in the system still keeps $\mathbf{B}$ the same. In this situation, Eq. (67) is not anymore time-reversible and therefore Eq. (64) must not be necessarily valid. More generally, reciprocity is known to be broken if we have:

- external magnetic field (see above),
- rotations of the system as a whole (since rotation moments $\mathbf{m}$ behaves pretty much like magnetic field),
- time dependence,
- nonlinearity.

C. z-reciprocity

Since our equations describe the evolution in z rather than in t, it is useful to reformulate the reciprocity condition also in z direction. We note that reciprocity is obviously invariant to the change of the the frame of reference, that is, if our cavity is reciprocal in one frame of reference, it is also reciprocal in the other frame of reference, because, if we reverse the direction of the evolution (time or z), the fact, that the system returned back to the initial conditions can not depend on the reference frame.

On the other hand, as it was mentioned in the previous section, explicit time dependence in the system can lead to nonreciprocity. Our situation, described by Eq. 1 of the main article, is obviously time dependent because of the time-dependent potential $V(t)$. Can this time dependent potential $V(t)$ lead to the nonreciprocity? In this section we show that this is not the case.

To start with, let us introduce "z-reciprocity" in analogy to time-reciprocity defined above. For this, we consider local, z-propagated evolution equation Eq. (29), which we repeat here for convenience:

$$\frac{\mathrm{d}a(z)}{\mathrm{d}z} = -\Gamma a(z) + \chi p(z). \tag{68}$$

We can repeat all the steps in Sec. III A. We start from taking $p = 0$ and calculating $a(z)$, which will be then $a(z) = a_0 e^{i\Gamma z}$. Then we propagate back, taking the radiated field as new $p(z)$. Now, we normalize $p(z)$ in such a way that

$$-\mathrm{d}W_{\mathrm{in}} = \mathrm{d}W_{\mathrm{out}} = |p|^2 \mathrm{d}z. \tag{69}$$

Note, that this normalization is different from the time-case Eq. (56). Next, we have, in analogy to Eqs. (59)-(60),

$$\frac{\mathrm{d}W_{\mathrm{in}}}{\mathrm{d}z} = -|p|^2,\ p(z) = \sqrt{2\Gamma} a_0 e^{-\Gamma z}, \tag{70}$$

and, analogously to Eq. (62), we have:

$$a(z) = -\chi e^{-\Gamma z} \int_0^z p(z') e^{\Gamma z'} dz' - a(0) e^{-\Gamma z} =$$

$$= -\frac{\sqrt{2\Gamma}\chi a_0}{2\Gamma} e^{-\Gamma z - \Gamma Z} \left(e^{2\Gamma z} - 1\right) + a_0 e^{-\Gamma z - \Gamma Z}, \tag{71}$$

where Z is the analog of T, that is, the "very big distance". The resulting reciprocity analog of Eq. (64) is the same as for the standard nonreciprocity:

$$|\chi| = \sqrt{2\Gamma}. \tag{72}$$

Note that if we have z-reciprocity, we must also have time-reciprocity and vise versa. This is because z- versus time description corresponds to the change of the reference frame from stationary to the moving with the soliton. As it was already mentioned above, if we make forward-time-backward-time cycle and our system returns to the initial state (the system is time-reversible), the same will happen if we do forward-z-backward-z (the system is z-reversible).

But can we have z-nonreciprocity? As mentioned in the main article, we can make the replacement $t \to x$, $z \to t$ in Eq. 1 of the main article, obtaining a fully traditional quantum-mechanical equation with the potential $V(x)$, obtained from $V(t)$ by the rule $V(t) \to V(x)$. Under this transformation, as is easy to see, z-reciprocity is transformed into time-reciprocity. Therefore, if our system were z-nonreciprocal, there would exist a potential $V(x)$ in standard quantum mechanics (by standard quantum mechanics we mean here the one described by Eq. 6 of the Methods section in the main article) that would lead to nonreciprocity. Since standard quantum mechanics does not possess such nonreciprocity, our system also cannot demonstrate z-nonreciprocity, and therefore cannot be time-nonreciprocal. In the next section, we present a careful numerical test supporting this general statement for our particular case.

D. Numerically testing reciprocity in the time-traps

As it was mentioned in the previous section, our system remains fully reciprocal. In particular, it remains

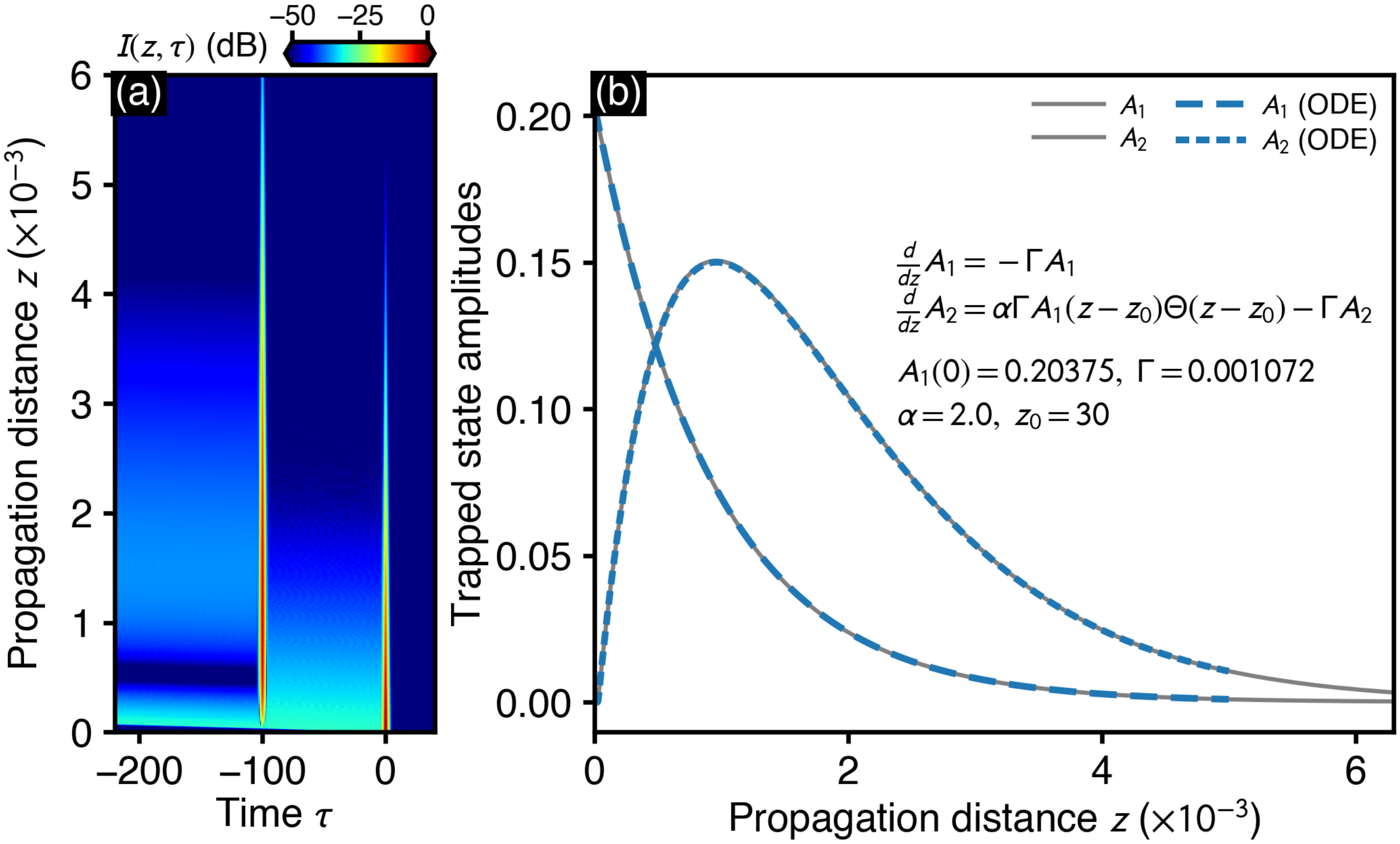

Supplementary Figure 1. Leakage from a linear time-trap and rescattering on another time-trap. (a) One trap (right) is pre-loaded with a pulse; The energy leaks it, traveling to the left and meeting the second trap (right), and loading it. Some energy, escaping both traps travels further to the left. (b) The amplitude of the state in the trap according to the Eq. 1 of the main text (gray solid lines) as well as according to the ordinary differential equations (ODEs) given in the figure (blue dashed curves) with the fit parameters α and Γ tuned to reproduce the dynamics of full linear equations in the best way. The units here are ps for time and nanometers for the distance

fully reversible; also, the energy if fully conserved (see Extended Fig. 2 of the main article).

Here we test the reciprocity numerically in our system. For this we consider, instead of loading our time-cavity with the ultrafast pulses (as we do in the main text), the process of slow leaking of the energy from the pre-loaded cavity as illustrated in Fig. 1. Here, we model fully linear situation (Eq. 1 of the main article), to exclude possible nonlinearities (which can break reciprocity). That is, instead of simulating time-trapping in the soliton, as we do in the main article, we simulate it in the pre-set time-potential $V(t)$, which has the sech-shaped form with the duration $\tau = 60$ fs. To test (non)reciprocity, we set up two such time-potentials $V(t)$ which are identical, but located on large distance $\tau_d = 100$ ps to each other. The energy, initially (at $z = 0$) stored in the right potential (see Fig. 1a) in the form of the trapped state, leaks, propagating to the left, meeting the left potential, partially trapped, partially transmitted. The reflected wave from the second (left) potential is negligible, since the well known property of sech potential to be reflectionless. The amplitudes of the trapped states in both potentials are shown in Fig. 1b, together with their fits by a set of ordinary differential equation (ODE) shown on the figure. These ODEs describe the dynamics of the amplitude of the first trapped state A_1, coupled to the dynamics of the amplitude in the second well A_2. As said, at $z = 0$ the first well is pre-loaded, and decay with the decay constant Γ. The emitted radiation achieves the second well (which is empty at $z = 0$) at the distance $z_0 = \tau_d/\beta_1$, where β_1 here is the inverse group velocity for the radiated wave. Observing this dynamics numerically, we can try to find, using the fit, the parameters Γ and α, determining the leakage rate from the both traps (which must be the same because the wells are identical) and the in-coupling coefficient for the second trap, respectively. This constellation allows to test the reciprocity directly in a single propagation scenario, without inverting z. Here we have obtained for α the best fit value $\alpha = 2.0$. This corresponds to the fully reciprocal behaviour. To see this, let's rewrite the equation for the second cavity as

$$\frac{\mathrm{d}A_2}{\mathrm{d}z} = -\Gamma A_2 + \chi p_2(z - z_0). \tag{73}$$

We define the furthermore pump for the second cavity

as $p_2(z) = p_1(z - z_0)$, where p_1 is the radiation, created by the first trap (escaped from it). Here the propagation of escaped radiation leads only to a delay, not a reshaping, reflecting the fact, that $p(z)$ is a very long pulse, which, because of its extraordinary duration does not experience modification due to higher-order dispersion. We furthermore assume $p_1(z) = Ge^{-\Gamma z}$, where G is yet unknown coefficient, which we obtain from the energy conservation. Namely, the energy inside the first cavity is $W_{\text{in}}^{(1)} = |A_1(z)|^2 = |A_1(0)|^2 e^{2\Gamma z}$, so that the energy change in the interval $[z, z+dz]$ is $dW_{\text{in}}^{(1)} = 2\Gamma |A_1(0)|^2 e^{2\Gamma z} dz$. On the other hand, the energy in the outgoing from the first cavity wave $W_{\text{out}}^{(1)} = \int_0^z |p_1(z)|^2 dz$, so that in the same interval $[z, z+dz]$ the energy change is $dW_{\text{out}} = |p_1(z)|^2 dz = G^2 e^{-2\Gamma z} dz$. From this, we readily obtain that $G = \sqrt{2\Gamma}|A_1(0)|$. Assuming furthermore for simplicity the real-valued positive $A_1(0)$ and taking the definition of p_2 above, this leads us to $p_2 = \sqrt{2\Gamma} A_1(z - z_0)$. Comparing this to Eq. (73) and to the equation for A_2 in Fig. 1b, we obtain that $\alpha = 2.0$ in Fig. 1b corresponds to $\chi = \sqrt{2.0 \times \Gamma}$, which denotes perfectly reciprocal situation Eq. (72).

That is, this numerical simulation confirms, that the dynamics in the trap, determined by Eq. 1 of the main article is fully reciprocal. This shows also that the "effective nonreciprocity," introduced in the main article is not "true nonreciprocity." To furthermore support the influence of the nonlocal memory effect ("nonlocal effective nonreciprocity") for the short pump pulses, we provided a further simulation shown in Fig. 2. There, we load the potential well (the same as in previous case) with a short pump pulse. In this case, the loaded amplitude A_1 can be calculated directly with the full equation Eq. 1 of the main text, or, alternatively, can be calculated via the local expression

$$\frac{\mathrm{d}A_2}{\mathrm{d}z} = \alpha A_1(z) - \Gamma A_2(z). \tag{74}$$

Here, the pump $A_2(z)$ is taken from the solution of the full equation Eq. 1, and we fit α and Γ in order to get the best result. For Γ, we have taken two models: in the first (denoted as M1 in the inset to Fig. 2), we assume $\Gamma = \text{const}$, whereas in the second case (denoted as M2) we assume $\Gamma = \Gamma_\infty + \gamma_0 e^{-\gamma_1 z}$. In the later case for $\tau = 60$ fs pump pule we obtained for the best fit $\alpha = 9.58982 \times 10^{-5}$, $\gamma_0 = 6.59588 \times 10^{-4}$, $\gamma_1 = 2.36225 \times 10^{-3}$. Most importantly, as the inset to Fig. 2 shows, if we repeat the fit for different pulse durations, we obtain, that the fit with $\Gamma = \text{const}$ works much worse than the fit with z-dependent Γ, indicating clearly that the constant Γ (corresponding to the local dynamics) is not the best approximation, and the nonlocality in the system is noticeable.

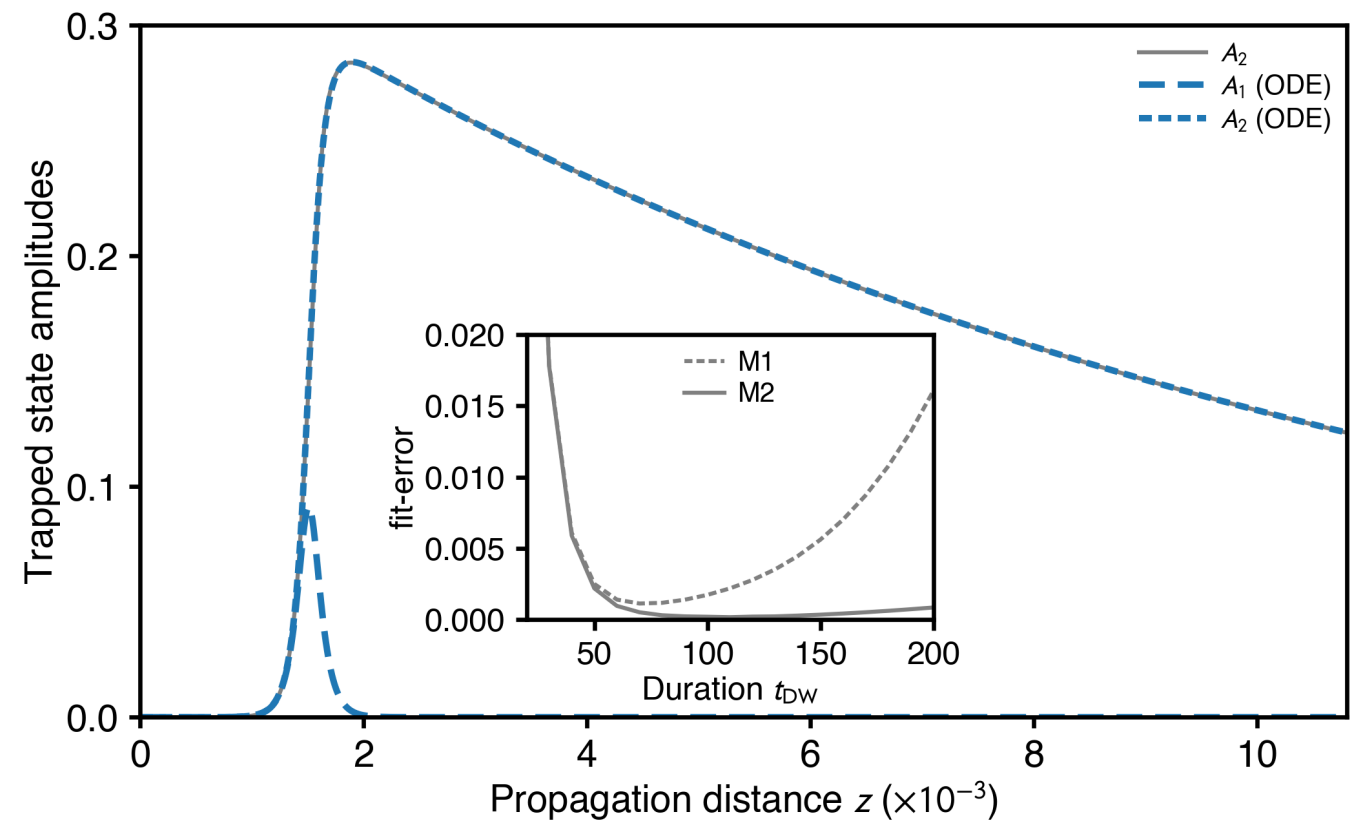

Supplementary Figure 2. Loading a time-trap with a short pulse and fitting with the model M1 and M2 (see text). The inset shows the dependence of fit error on the pulse duration.

IV. SINGLE-PHOTON QUANTUM WAVEPACKET PROPAGATION: EXTENDED DISCUSSION

We consider the stochastic Schrödinger equations derived in [2, 3]: (here including Raman nonlinearity):

$$\partial_z A_\omega = i\beta(\omega) A_\omega + i\frac{\tilde{\gamma}^*}{2} X_\omega + i\frac{\tilde{\gamma}^*}{2} Y_\omega + i\tilde{\gamma}^* N_\omega, \tag{75}$$

where

$$X_t = (C_t^* B_t + \sigma_t^{(1)}) B_t, \tag{76}$$

$$Y_t = (B_t^* C_t + \sigma_t^{(2)}) C_t, \tag{77}$$

$$N_t = \left(\int_0^\infty (R_\tau - \delta(\tau)) |B_{t-\tau}|^2 d\tau + \sigma^{(3)} \right) B_t, \tag{78}$$

$X_\omega = \mathcal{F}[X_t]$, $Y_\omega = \mathcal{F}[Y_t]$, $N_\omega = \mathcal{F}[N_t]$, $\tilde{\gamma} = (\omega_0 + \omega) r_\omega$, $r_\omega = (\gamma(\omega)/(\omega_0 + \omega))^{1/4}$, $B_\omega = r_\omega A_\omega$, $C_\omega = r_\omega^* A_\omega$, $\sigma_t^{(i)}$ ($i = \{1, 2, 3\}$) are stochastic processes with the correlations given by (in frequency domain):

$$\langle \sigma_\omega^{(1)} \sigma_{\omega'}^{(1)} \rangle = -2i\frac{\hbar}{T}\delta_{\omega\omega'}, \tag{79}$$

$$\langle \sigma_\omega^{(2)} \sigma_{\omega'}^{(2)} \rangle = -i\frac{\hbar}{T}(R_\omega - 1 + ig_\omega)\delta_{\omega\omega'}, \tag{80}$$

$$\langle \sigma_\omega^{(3)} \sigma_{\omega'}^{(3)} \rangle = -\frac{\hbar}{T} g_\omega \delta_{\omega\omega'}, \tag{81}$$

$g_\omega = 2\mathrm{Im}\,(R_\omega)(H(\omega) + n_\omega)$, $R_\omega = \int_{-\infty}^{+\infty} R(t) e^{i\omega t} dt$, $R(t)$ describes both the Raman and Kerr response, $H(\omega)$ is the Heaviside step function and $n_\omega = 1/(e^{\hbar\omega/k_b T} - 1)$, with k_b being the Boltzmann constant and T the temperature of the waveguide.

Our first goal is to estimate the direct, immediate action of noise on the trapped state, leading to the decoherence of the photon. As the second step, we note

that the quantum noise leads to one more additional effect - fluctuating of the trap. Finally, we show that on the propagation distances where all the above mentioned decoherence and loss terms are negligible, the single-photon wavepacket described by $\Psi(t)$ (see Methods and also the paragraphs below for definitions of the denotations) obeys Eq. 1 of the main article.

So, we start with the estimation of the decoherence action of the photons. In our case, we consider Raman-free response. In this situation $R_t = \delta(t)$, $R_\omega = 1$ and therefore $g_\omega = 0$ so that the noise contributions $\sigma^{(2)}$ and $\sigma^{(3)}$ disappear. Besides, for the sake of simplicity, since our goal is the order of magnitude estimation, we assume $\gamma(\omega) = \mathrm{const}(\omega) \approx \gamma_0$. Normalizing the propagation distance to the nonlinear length of the single photon of frequency ω_0 in a waveguide $L_{\mathrm{NL},1} = 1/(\gamma_0 P_1)$, where $P_1 = \hbar\omega_0/\mathcal{T}$ is the power created by a single photon "distributed" homogeneously over the whole time $\mathcal{T}$. Besides, we normalize A_t as $A_t \to A_t/\sqrt{P_1}$. After those manipulations we obtain:

$$\partial_z A_t = i\beta(\omega)A_t + i(|A_t|^2 + \sigma'_t)A_t, \tag{82}$$

where now $\langle \sigma'_t \sigma'_{t'} \rangle = 4\pi^2 L_{\mathrm{NL},1}/(\mathcal{T}^2\omega_0)\delta_{tt'}$ and $\beta(\omega)$ is correspondingly re-normalized. In the normalization above $|A_t|^2 = n(t)$ describes the average photon density $n(t)$ at time t.

If we furthermore, for the sake of simplicity of estimation, disregard the temporal dependence, we will obtain

$$\partial_z A = i(n + \sqrt{2\chi'}\sigma''_z)A, \tag{83}$$

where we recast the temporal noise into spatial noise, converting "time" into "propagation distance" by re-normalizing the noise term by the group velocity v_g, this obtaining $\langle \sigma''_z \sigma''_{z'} \rangle = \delta_{zz'}$, $\chi' = 2\pi^2 L_{\mathrm{NL},1}/(\mathcal{T}^2\omega_0 v_g)$. This equation describes an oscillator with fluctuating frequency [4]. The correlation function $\langle A_z A^*_{z'} \rangle \propto \exp\left(in(z-z') - \chi'|z-z'|\right)$, which describes decoherence on the length scale (taking into account normalization of z) $L_{\mathrm{decoh}} = L_{\mathrm{NL},1}/\chi'$ which, for the parameters used here, is $L_{\mathrm{decoh}} \approx 10^5$ km, is much larger than the distance at which the photon is lost due to the linear losses. Therefore, the decoherence can be well neglected. We note that the possible noise terms induced by Raman $\sigma^{(2)}$ and $\sigma^{(3)}$, as it is seen from Eqs. (75)-(81), have the same order of magnitude and therefore also negligible.

On the other hand, if we substitute $A_t = u(t) + \Psi(t)$ into Eq. (82), after separating terms we will obtain equations for u and Ψ:

$$\partial_z u = i\beta(\omega)u + i|u|^2 u + \sigma'_t u, \tag{84}$$

$$\partial_z \Psi(t) = i\beta(\omega)\Psi(t) + i|u|^2 A_t, \tag{85}$$

where we neglected the terms of the second order in Ψ and σ' such as for instance $\sigma'\Psi$. The solution u in this case is noisy and can be represented as $u = u_0 + \sigma_s$, where u_0 represents the solution of Eq. (84) with $\sigma'_t = 0$. In Eq. (85) this leads to (neglecting the terms with second order of σ_s):

$$\partial_z \Psi(t) = i\beta(\omega)\Psi(t) + i|u_0|^2 A_t + 2i\mathrm{Re}\left(u_0\sigma_s\right)\Psi(t), \tag{86}$$

If we assume no noise ($\sigma_s = 0$) in Eq. (86), the resulting equation is equivalent to Eq. 1 of the main article. The term $\sim u_0\sigma_s\Psi(t)$ describes the action of the fluctuations of the photon number in the soliton on the wavepacket $\Psi(t)$. The amplitude of this term is $\approx \sqrt{n_s}$, where n_s is the mean number of photons in the solitons. For the parameters we used here, we have $n_s \approx 10^8$ photons in the soliton. Taking into account the normalization used in the article, the corresponding dephasing length is then $L_{\mathrm{decoh}} = L_{\mathrm{NL},1}/\sqrt{n_s} \approx 10$ km, which is much smaller than the decoherence length calculated in the previous paragraphs but still the order of magnitude larger than the photon's linear loss length even in good waveguides.

Putting all the results together, we see that the trapped state experiences decoherences on the scale of tens of kilometers, assuming a "typical" waveguide and a short soliton with the duration of order few femtoseconds and the number of photons $n_s \approx 10^9$, we will obtain the corresponding losses and dephasing terms on the 10-kilometer range or more, that is, of the same order of magnitude as the linear losses. That is the corresponding quantum noise-induced term do not decrease the trapping quality.

Finally, suppose that the initial full state (soliton + weak radiation) is the sum of both: $\rho = \rho_s + \rho_\Psi$, meaning that there no correlations between the soliton and trapped state at the entrance of the waveguide $z = 0$. Assuming that $u(\omega) = \mathcal{F}[u(t)]$ and $\Psi(\omega) = \mathcal{F}[\Psi(t)]$ are well separated from each other (that is, their spectra have a negligible overlap), and taking into account that, according to the said before, the decoherence terms can be neglected, we obtain that the weak radiation part of the wavepacket remains disentangled from the photonic state, and can be described by a pure state for $z > 0$. Denoting this state as $\rho_\Psi = |\Psi\rangle\langle\Psi|$ and assuming that $|\Psi\rangle$ represents a single photon wavepacket: $|\Psi\rangle = \sum_\omega \Psi(\omega)a^\dagger_\omega$, the consideration above shows that this single-photon wavepacket remain such for $z > 0$.

Furthermore, Eq. (86) (and Eq. 1 of the main article) describes the evolution of the coherent amplitudes in the positive P-decomposition $P(\Psi, \Psi^*)$ of ρ_Ψ. Since Ψ can be arbitrary small number and for a small Ψ the leading term in a weak coherent state $|\alpha_\Psi\rangle$ is $|0\rangle + \int \Psi(\omega)\,|1\rangle_\omega\, d\omega$, we must conclude that $\Psi(\omega)$ for the Fock state with number of photons equal to one is also governed by the same equation. In the other words, we conclude that $\Psi(\omega)$ in ρ_Ψ must be the same as quantity as $\Psi(\omega)$ in Eq. 1 of the main article, that is, Eq. 1 of the main article describes the evolution of the single-photon wavepacket (as soon as noise terms are neglected).

SUPPLEMENTARY REFERENCES

[1] Hermann A Haus, *Electromagnetic noise and quantum optical measurements* (Springer Science & Business Media, 2000).
[2] Juan Bonetti, SM Hernandez, and DF Grosz, "Master equation approach to propagation in nonlinear fibers," Opt. Lett. **46**, 665–668 (2021).
[3] J Bonetti, DF Grosz, and SM Hernandez, "Quantum noise in fibers with arbitrary nonlinear profiles," Phys. Rev. Lett. **126**, 213602 (2021).
[4] Crispin W Gardiner and C Gardiner, *Stochastic methods: a handbook for the natural and social sciences*, Vol. 4 (Springer Berlin, 2009).
