# Supplementary material for: Photon trapping in a time cavity flying at the speed of light
Source: Nat Commun. 2026 Aug 29;17:9235. doi: 10.1038/s41467-026-77059-1 (PMC13526024; doi:10.1038/s41467-026-77059-1)
Supplement: Supplementary file 2 [file 41467_2026_77059_MOESM2_ESM.pdf]

nature portfolio

Peer Review File

Photon trapping in a time cavity flying at the speed of light

Corresponding Author: Dr Ihar Babushkin

This manuscript has been previously reviewed at another journal. This document only contains information relating to versions considered at Nature Communications.

This file contains all reviewer reports in order by version, followed by all author rebuttals in order by version.

Attachments originally included by the reviewers as part of their assessment can be found at the end of this file.

Version 0:

Reviewer comments:

Reviewer #1

(Remarks to the Author)
See PDF

(Remarks on code availability)

Reviewer #2

(Remarks to the Author)
I thank the authors for their thorough revision of the manuscript and for addressing the comments thoughtfully and constructively.

Comment #2
The authors have adequately addressed this comment in the revised manuscript. In particular, they introduced new sections entitled “Linear Losses for the Trapped States” and “Dissipation Due to Waveguide Imperfections”, and provided a detailed derivation of the corresponding equations, including an analogue of the Fermi Golden Rule in the Supplementary Material (Sec. II). These additions clarify the physical origin of losses and strengthen the theoretical framework. They covered my concern very well!
I do not have further concerns regarding this point.

Comment #3
The authors clarify the physical origin of the memory effect and provide additional discussion and examples illustrating its role in energy loading. While the comparison with established quantum memory schemes remains qualitative, the revised presentation sufficiently distinguishes the proposed mechanism from slow-light and cavity-based approaches.

In summary, the authors have adequately addressed my comments, and the revisions—including new sections and expanded discussions—have substantially strengthened the manuscript. I therefore recommend publication in Nature Communications.

(Remarks on code availability)

Version 1:

Reviewer comments:

Reviewer #1

(Remarks to the Author)
The authors have made considerable changes to the manuscript as a response to my comments. I find the paper interesting, and am happy to move forward with recommending publication.

As a small remark, the newly added Fig. 4e has different fonts than the rest of the figure.

(Remarks on code availability)

Open Access This Peer Review File is licensed under a Creative Commons Attribution 4.0 International License, which permits use, sharing, adaptation, distribution and reproduction in any medium or format, as long as you give appropriate credit to the original author(s) and the source, provide a link to the Creative Commons license, and indicate if changes were made.
In cases where reviewers are anonymous, credit should be given to 'Anonymous Referee' and the source.
The images or other third party material in this Peer Review File are included in the article's Creative Commons license, unless indicated otherwise in a credit line to the material. If material is not included in the article's Creative Commons license and your intended use is not permitted by statutory regulation or exceeds the permitted use, you will need to obtain permission directly from the copyright holder.
To view a copy of this license, visit https://creativecommons.org/licenses/by/4.0/

Dear Editor,

Please find enclosed the revised manuscript NCOMMS-25-75130-T, which has been updated according to the comments and suggestions of Reviewer #1. We note that the second of the two reviewers, Reviewer #2, expressed the full satisfaction with the revisions made in the previous version of the manuscript, and recommended it for publication; we sincerely thank Reviewer #2 for their fully positive assessment.

Below, we provide detailed responses to the comments of Reviewer #1, along with a description of the changes made to the manuscript. All modifications are also highlighted in the redline version submitted alongside this revision.

Answer to Reviewer #1

Comment # 1

The authors largely disagreed with my previous suggestions, and have not made substantial changes based on my feedback. As a result, many of my existing opinions still hold. Most importantly, I still believe that the authors over-emphasize the novelty of claims related to TBP breaking (and its implications for energy storage). Secondarily, I still still believe that the quantum description of the system for single photons follows quite immediately from the linear wave equation for trap states, as I described previously.

Correspondingly, I find that the manuscript is still generally not clear, and thus has a high risk of overclaiming. For example, one of the first paragraphs of the body of the text states: "Our list of unusual features of time-trapping begins with the observation that it can exhibit effectively zero radiation through the walls." I still do not find this to be a novel point, and find that it is already clear from the prior knowledge of trap states. I find the results derived from the coupled mode theory description for loading/unloading leaky trap states to be much more interesting.

Our Answer:

The Reviewer found the parts related to loading and unloading more interesting. In response, we have noticeably shifted the focus of the presentation toward these aspects; three of the five figures in the main text are now devoted to them.

We appreciate the Reviewer's point that some of our statements may have appeared overstated. In the revised version of the manuscript, we have added detailed clarifications to avoid any impression of overstatement.

In particular, we now explicitly mention in the manuscript that the breaking of the TBP limit may indeed appear rather trivial, given the short duration of optical solitons. However, regardless of how trivial it may seem, TBP breaking clearly highlights the dramatic difference between time traps and traditional cavities, since in conventional cavity physics the TBP represents a fundamental — and perhaps even unbreakable — limitation. We also emphasize that the TBP is not a property of the trapped state alone, but rather of the combined system consisting of the trapped state and the coupling channel. This is why we discuss it in the context of loading and unloading.

Furthermore, regarding zero losses through the walls (ZLW), in order to avoid overstatement, we now explicitly acknowledge in the revised manuscript that ZLW are, formally, already contained in the equations for trapped states derived previously (independently by several authors), through the appearance of purely real rather than complex eigenvalues. However, this important fact appears to have remained fully unnoticed before. Alternatively, one might even wonder whether it is merely a mathematical artefact of the equations. We not only bring ZLW to light, but also elucidate the physical mechanism behind them. We believe that these results — ZLW and TBP breaking — are particularly interesting for the community working on traditional ultrahigh-Q cavities, whereas other aspects of the work, such as loading and unloading, may be more appealing to the soliton and ultrashort-pulse community. Since these two communities — ultrahigh-Q cavity physics and ultrashort-pulse physics — are largely disjoint, we hope that the present paper provides an interesting common ground between them.

Regarding the description of single-photon Fock states, we have also reformulated the presentation to avoid any possible impression of overstatement. We now state explicitly that the equivalence of the equations governing the single-photon wave shape $\Psi(t)$ and the weak classical electric field $E(t)$ (provided that noise and decoherence are neglected) may be viewed as a direct consequence of quantum–classical correspondence and, in this sense, may appear somewhat trivial. Nevertheless, such a description i) has, to the best of our knowledge, not appeared previously in this or a similar context, and ii) renders the treatment of quantum Fock states significantly simpler than the operator-based approaches that are currently predominant in quantum nonlinear optics. Moreover, our single-photon treatment can in many cases be extended straightforwardly to nonclassical multiphoton states. In the revised manuscript, we now include an example of multiphoton statistics in Fig. 4e (see also Comment #2 below).

Comment # 2

Furthermore, I feel that the way the manuscript is laid out (in terms of writing and figures) does not serve the work as well as it could. There are a lot of topics, and I find it a bit disjointed. I provide a few examples here, with the intention of aiding the manuscript toward publication and increasing the impact of the authors' work:

- *Fig. 1 dedicates a lot of space to the basic description of trap states in solitons, which are already known; and Fig. 2 has a schematic-level description of spatial versus temporal trapping. This is all good content, but to me it feels very far down in the paper before I get to something that feels like a concrete result.*

- *Fig. 3 feels dense in a way that is somewhat overwhelming (to me at least...). There are many similar looking plots, and the overall point (about using mode beating to temporally shape pulses) is hard to identify visually. I think a less dense version could be more impactful.*

- *Fig. 4 is potentially the most important in the paper, in terms of the authors' primary claim for novelty of physics in this system. As mentioned above, I feel that this appears a bit far down in the paper. I think it could be helpful to have even more explicit quantitative plots showing what their system vs a normal trap can do.*

- *There is time spent in the text describing the quantum treatment of the system for single photons, but this description is not used to present a result shown in a figure.*

Our Answer:

In the revised version of the manuscript, we have implemented the Reviewer's suggestions through the following changes:

- The Reviewer found Figs. 1 and 2 superficial. Therefore, we have removed Fig. 2 from the main text and placed it after the manuscript as an Extended Figure. The related discussion in the main text has also been substantially shortened. We cannot remove the introductory figure (Fig. 1) entirely, as this would clearly be poor style. However, we have simplified it by removing the dynamics of the trapped state (former Fig. 1f).
- We are pleased that the Reviewer appreciates the set of results presented in the former Fig. 4. In the revised version, we have split this figure into two for greater clarity of presentation. These figures now also appear somewhat earlier in the manuscript, namely as Figs. 3 and 4. In addition, as suggested by the Reviewer, we have added curves (Fig. 3d, gray lines) showing the best achievable behavior of a traditional reciprocal cavity with the same decay rate and the same input pulse duration.
- Following the Reviewer's suggestion, we simplified Fig. 3 (now Fig. 2) by removing panels (d) and (g). On the other hand, in response to the Reviewer's request for a stronger presence of quantum optics, we added the Wigner functions (see more details in the next paragraph).
- To strengthen the quantum-optical part of our presentation, as requested by the Reviewer, we have added two new elements:

  i) In Fig. 3(e,g), we introduced the Wigner functions of the single-photon Fock states. In particular, the Wigner functions exhibit negative regions, indicating the genuinely nonclassical character of the Fock-state evolution. Note that the Wigner function corresponding to a weak classical wave would remain nonnegative (see the revised Methods section, "Visualization of the Dynamics: Spectrogram and Wigner Function", for more details). In addition, in the revised manuscript we now also discuss the resulting entanglement, analogous to the entanglement generated when a single photon is sent through a beam splitter, but here involving time-modes instead.

  ii) To illustrate the quantum-optical consequences of the effective nonreciprocity, we added Fig. 4e. In this figure, we show the photon statistics inside the time trap that emerge after many independent single-photon Fock states are sent into the trap sequentially. The state formed inside the trap during this process is a multiphoton state with photon statistics impossible to achieve in a traditional cavity (assuming the same parameters of the cavity and excitation). We also note that the manuscript discusses photonic memories in the paragraph preceding this result.

Comment # 3

One technical question:

- *When discussing time trapping, the authors focus on the energy fraction of an incoming pulse that can be loaded into the trap. One of the main reasons that standard cavities are used is to enhance the effective field strength of light inside the cavity compared to outside it. Can the authors comment on how this behavior looks for time trapping? Intuitively, I might expect that these time traps have a more "gentle" effect on the incoming pulse; they may effectively capture a large bandwidth signal, but might not enhance the signal very much.*

Our Answer:

Enhancement factor is indeed a very important property. In our situation, however, it should be considered with some care, since it is conventionally defined for continuous or very long pump pulses, or, alternatively, for periodically repeated pump pulses, whereas here we mainly consider a single ultrashort pump pulse. Nevertheless, even in such a situation, the enhancement factor can of course be defined. In the case presented in Fig. 4, the enhancement factor (if defined as the ratio of the intensities inside and outside the cavity) already reaches approximately 3.0 for a 60 fs input pulse. Although this number may appear modest, one should keep in mind that we are considering an ultrafast excitation driven by a single excitation pulse. This value is about a factor of five larger than what is fundamentally achievable in conventional cavities with the same parameters for input pulses of identical duration. Indeed, the enhancement factor X is proportional to the energy stored in the cavity, $X \sim \epsilon$, and thus, in agreement with Eq. 4, $X \sim N$. Therefore, the enhancement in the cavity factor is directly linked to the advantage in the effective nonreciprocity. We discuss this point in the revised manuscript in the paragraphs following Eq. 4.

Another situation (in which the enhancement factor becomes especially meaningful) is, as mentioned above, when many pump pulses are sent sequentially and periodically in time. In this case, the energy in the trap grows until saturation is reached. In this regime, one can speak about enhancement in a more traditional sense. If we compare a traditional cavity and a time-trap with identical decay times, the above-mentioned enhancement, $X \sim N$, remains present in this situation as well.

Comment # 4

Small things:

- *Fig. 3g is not mentioned in the caption*
- *Fig. 4d is labeled "Nornal trap"*

Our Answer:

We would like to thank the Reviewer for noticing these typos. We have corrected them, along with several additional typographical errors. (Note that the former Fig. 3g is no longer present in the revised version, as discussed above.)

Dear Editor,

We would like to thank the Reviewer for the positive assessment of our work.

In the only comment, the Reviewer asked us to change the font in Fig. 4e (now Fig. 6e, following the renumbering of figures requested in the checklist; see the Cover Letter for details). We thank the Reviewer for this remark and have changed the font accordingly.

Sincerely yours,
on behalf of coauthors,
Ihar Babushkin.

I have reviewed the paper *"Photon trapping in a time cavity flying at the speed of light"* by authors Babushkin, Melehert, Morgner, and Demircan for a third time, now for *Nature Communications*. There are potentially interesting theoretical results here, and I strongly believe that there is a possible path for the authors to turn their results into a strong and clear paper. In order to recommend acceptance, I would want to see substantial adjustment of the paper's claims and presentation.

The authors largely disagreed with my previous suggestions, and have not made substantial changes based on my feedback. As a result, many of my existing opinions still hold. Most importantly, I still believe that the authors over-emphasize the novelty of claims related to TBP breaking (and its implications for energy storage). Secondarily, I still still believe that the quantum description of the system for single photons follows quite immediately from the linear wave equation for trap states, as I described previously.

Correspondingly, I find that the manuscript is still generally not clear, and thus has a high risk of overclaiming. For example, one of the first paragraphs of the body of the text states: *"Our list of unusual features of time-trapping begins with the observation that it can exhibit effectively zero radiation through the walls."* I still do not find this to be a novel point, and find that it is already clear from the prior knowledge of trap states. I find the results derived from the coupled mode theory description for loading/unloading leaky trap states to be much more interesting.

Furthermore, I feel that the way the manuscript is laid out (in terms of writing and figures) does not serve the work as well as it could. There are a lot of topics, and I find it a bit disjointed. I provide a few examples here, with the intention of aiding the manuscript toward publication and increasing the impact of the authors' work:

- Fig. 1 dedicates a lot of space to the basic description of trap states in solitons, which are already known; and Fig. 2 has a schematic-level description of spatial versus temporal trapping. This is all good content, but to me it feels very far down in the paper before I get to something that feels like a concrete result.
- Fig. 3 feels dense in a way that is somewhat overwhelming (to me at least…). There are many similar looking plots, and the overall point (about using mode beating to temporally shape pulses) is hard to identify visually. I think a less dense version could be more impactful.
- Fig. 4 is potentially the most important in the paper, in terms of the authors' primary claim for novelty of physics in this system. As mentioned above, I feel that this appears a bit far down in the paper. I think it could be helpful to have even more explicit quantitative plots showing what their system vs a normal trap can do.
- There is time spent in the text describing the quantum treatment of the system for single photons, but this description is not used to present a result shown in a figure.

One technical question:

- When discussing time trapping, the authors focus on the energy fraction of an incoming pulse that can be loaded into the trap. One of the main reasons that standard cavities are used is to enhance the effective field strength of light inside the cavity compared to outside it. Can the authors comment on how this behavior looks for time trapping? Intuitively, I might expect that these time traps have a more "gentle" effect on the incoming pulse; they may effectively capture a large bandwidth signal, but might not enhance the signal very much.

Small things:

- Fig. 3g is not mentioned in the caption
- Fig. 4d is labeled "Nornal trap"
